# Perinatal morphine exposure induces chromatin and transcriptomic remodeling to alter immune and metabolic function

**DOI:** 10.3389/fimmu.2026.1835359

**Published:** 2026-07-06

**Authors:** Julia R. Ferrante, Yanmiao Du, Xin Zhang, Jacob D. Neice, Wei Wang, Chang Lu, Julie A. Blendy

**Affiliations:** 1 Department of Systems Pharmacology and Translational Therapeutics, Perelman School of Medicine, University of Pennsylvania, Philadelphia, PA, United States; 2 Department of Chemistry and Biochemistry, University of California, San Diego, San Diego, CA, United States; 3 Department of Chemical Engineering, Virginia Tech, Blacksburg, VA, United States; 4 Department of Cellular and Molecular Medicine, University of California, San Diego, San Diego, CA, United States

**Keywords:** immune, metabolism, morphine, neonatal opioid withdrawal syndrome, omics, opioid, sequencing, withdrawal

## Abstract

**Introduction:**

Infants exposed to opioids in utero are at risk of developing Neonatal Opioid Withdrawal Syndrome (NOWS). Rodent models of perinatal opioid exposure can reliably recapitulate the acute withdrawal and developmental deficits exhibited in clinical NOWS, but few persisting phenotypes are consistently observed between studies, limiting mechanistic insight into the long-lasting effects of early-life opioid exposure.

**Methods:**

To investigate the enduring impact of perinatal opioid exposure, we employed a multi-region, multi-omic approach. We combined RNA sequencing (RNA-seq) and H3K27ac chromatin immunoprecipitation sequencing (ChIP-seq) from NeuN+ neuronal nuclei in a mouse model of NOWS. Additionally, cytokine levels were measured in the brain and spleen, and physiological responses were assessed under both basal and immune-challenged conditions.

**Results:**

Analysis revealed differentially expressed genes and H3K27ac modifications were enriched for immune and metabolic pathways in hypothalamic neurons. Transcription factor network inference identified state-dependent rewiring of immune and metabolic regulatory circuits, with Transcription factor 4 (TCF4) emerging as a convergent epigenomic and transcriptional hub under immune-challenged conditions. Consistent with molecular signatures, cytokine levels were suppressed in morphine-exposed mice both in adulthood, both at baseline and under immune-challenged conditions. Several metabolic properties, including changes in weight and basal body temperature, were also altered.

**Discussion:**

Our findings suggest that perinatal opioid exposure creates a lasting enhancer imprint in hypothalamic neurons, leading to suppressed baseline immune gene expression and altered regulatory responses to subsequent inflammatory challenges. These epigenomic and transcriptional changes may underlie the long-term physiological impacts of early-life opioid exposure, offering new insights into the enduring consequences of NOWS.

## 1 Introduction

The ongoing opioid epidemic in the United States carries additional public health considerations for pregnant women and their offspring. In 2018, one in six women of reproductive age were prescribed an opioid (1). When infants are exposed to opioids *in utero*, they are at risk of developing neonatal opioid withdrawal syndrome (NOWS), which occurs upon sudden cessation of opioids after birth and is characterized by tremors, irritability, feeding difficulty, and respiratory dysfunction (2). These acute withdrawal symptoms typically arise within 24–72 hours after birth (3, 4). Recent studies estimate that rates of NOWS diagnoses have increased approximately 82% from 2010-2017, representing an all-time high in incidence of disease (5, 6).

The long-term developmental consequences of perinatal opioid exposure remain poorly understood. Clinical studies have found persisting cognitive, behavioral, and physical deficits in humans, but rodent models have been unable to consistently replicate these findings, making it difficult to investigate underlying molecular mechanisms (7, 8). The variability in experimental outcomes is heavily influenced by methodological differences (including opioid type, exposure duration, and administration route), and many models fail to produce robust adult phenotypes altogether (9). Furthermore, research has been primarily focused on attempting to characterize a small set of behaviors, such as alterations in affect and reward, leaving other potential phenotypes unexplored. Newer studies have proposed an emerging hypothesis that perinatal opioid exposure alone may not cause overt deficits but instead creates a latent vulnerability environment in which phenotypes only arise upon exposure to a secondary stressor later in life (10, 11).

Transcriptomic changes and epigenetic marks, such as histone modifications, provide a molecular memory of developmental exposures (including opioids), which can influence gene expression long after the insult is removed (12, 13). *In vitro* studies and adult rodent models have shown that opioids change histone conformation through induction of acetyltransferase, leading to widespread changes in chromatin accessibility (14). Despite this, no studies have systematically profiled enhancer acetylation following perinatal opioid exposure specifically, resulting in a major gap in the understanding of the molecular underpinnings of opioid-induced developmental reprogramming.

We employed a hypothesis-generating approach to characterize novel phenotypes by leveraging epigenetic information taken from our mouse model of NOWS to identify pathways that may produce relevant molecular and behavioral outcomes. Beginning with a high-level combination of methods for screening, including a novel assay of microfluidic oscillatory washing chromatin immunoprecipitation (MOWChIP-seq (15–17)), we identified brain-wide transcriptomic changes and mapped histone modifications induced by perinatal opioid exposure. Regions surveyed included those critical for cognition and stress regulation (prefrontal cortex, hippocampus), reward (striatum), and homeostatic control (hypothalamus). As the hypothalamus displayed the greatest number of histone modifications, we proceeded with additional studies targeted towards this region. We uncovered significant enrichment in hypothalamic immune and metabolic pathways, prompting us to probe for related, latent molecular and behavioral phenotypes in morphine-exposed mice.

## 2 Results

### 2.1 Atlas-guided integration of chromatin and transcriptional data reveals region- and condition-specific regulatory states

**Figure 1:**
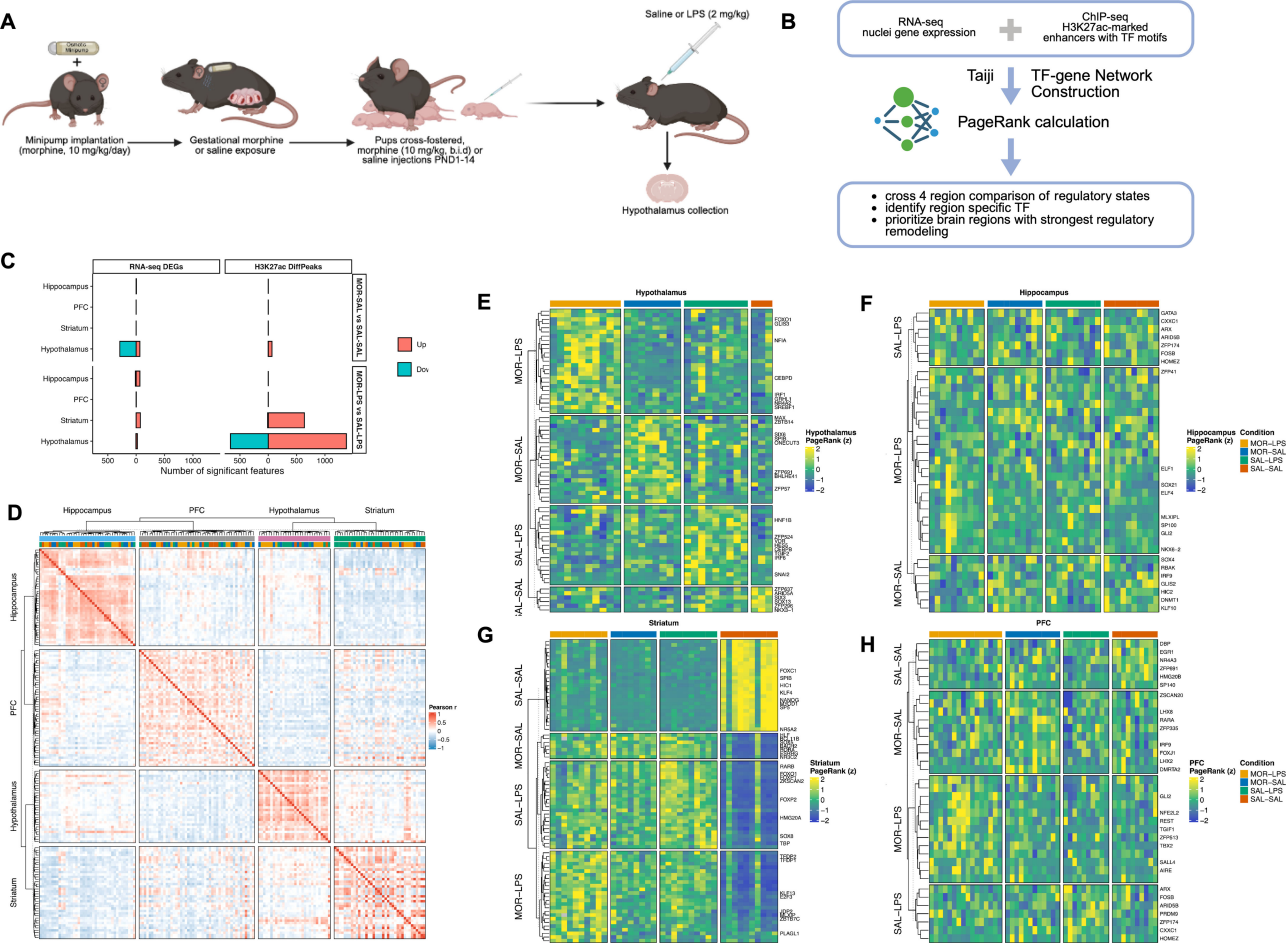
Experimental design and atlas-guided inference of transcription factor regulatory states across brain regions. **(A)** Schematic of the perinatal morphine exposure paradigm. Female mice received continuous morphine (10 mg/kg/day) or saline via osmotic minipump throughout gestation. Offspring were cross-fostered at birth and received twice-daily morphine or saline injections from postnatal day (PND) 1–14. In adulthood, mice were challenged with saline or lipopolysaccharide (LPS; 2 mg/kg) prior to tissue collection. **(B)** Overview of the integrative transcription factor (TF) activity inference framework. RNA-seq expression profiles and H3K27ac ChIP-seq–defined regulatory elements annotated with TF motif information were integrated using Taiji to construct weighted TF–gene regulatory networks. TF influence was quantified by PageRank, enabling cross-region and cross-condition comparisons of regulatory states. **(C)** Summary of differential features identified across brain regions. Bar plots show the number of significantly altered RNA-seq genes and H3K27ac peaks (enhancers and promoters) in hippocampus, prefrontal cortex (PFC), striatum, and hypothalamus. **(D)** Sample-level similarity of transcriptional and epigenomic profiles across regions. Heatmap displays Pearson correlation coefficients between samples based on combined RNA-seq and H3K27ac features, highlighting strong region-specific structure. **(E–H)** Region- and condition-specific transcription factor programs. Heatmap summarizing group-specific transcription factors (GS-TFs) across brain regions and experimental conditions, defined by condition-enriched TF PageRank activity. Limited overlap between regions indicates distinct regulatory programs, with the hypothalamus exhibiting prominent condition-dependent TF specialization.

Mice were exposed to morphine throughout gestation and for the first two postnatal weeks, as previously described (18) (**Figure 1A**). To enable systematic comparison of molecular responses across brain regions, we integrated RNA-seq and H3K27ac ChIP-seq data from hypothalamus, hippocampus, PFC, and striatum using transcription factor (TF) regulatory network inference (**Figure 1B**). This integrative framework combines chromatin-defined regulatory elements with gene expression to infer TF activity and facilitates comparison of regulatory states across regions and experimental conditions. All RNA-seq and H3K27ac ChIP-seq libraries were generated from FACS-isolated NeuN+ neuronal nuclei, providing a neuron-enriched molecular profile of each region; glial contributions are not captured in these datasets.

Sample-level correlation analysis revealed strong region-specific structure across molecular modalities, indicating that brain region represents the dominant axis of variation across samples, despite identical exposure paradigms and immune challenge (**Figure 1D**). Consistent with this regional structure, the number and balance of molecular changes differed between tissues. Under basal conditions, RNA-seq identified more differential features than H3K27ac peak changes across all regions, whereas immune challenge was associated with widespread enhancer-level remodeling accompanied by comparatively modest transcriptional output (**Figure 1C**). These patterns were not uniform across regions; hippocampus, PFC, and striatum showed either limited chromatin changes or relatively small transcriptional responses, while the hypothalamus exhibited a distinct distribution of differential RNA-seq and H3K27ac features across conditions. As such, we chose the hypothalamus as a specific region of interest to examine transcription factor (TF) regulatory influence, and as a focus for additional molecular and behavioral studies.

Using Taiji, TF activity was inferred as PageRank scores derived from region-specific TF–gene regulatory networks integrating RNA-seq expression and H3K27ac regulatory information. To compare TF activity patterns across tissues and conditions, group-specific transcription factors (GS-TFs) were defined based on condition-enriched TF PageRank activity, and TF activity was visualized as PageRank z-scores across samples within each brain region (**Figures 1E–H**). These region-resolved heatmaps revealed condition-dependent TF activity patterns across all four regions, with limited overlap in GS-TF activity between tissues, consistent with region-specific regulatory profiles. Among the regions examined, hypothalamic samples displayed particularly clear separation of TF activity patterns across MOR–SAL, SAL–LPS, and MOR–LPS conditions relative to other regions (**Figures 1E–H**), further motivating focused analysis of hypothalamic regulatory changes in subsequent sections.

Together, these results show that atlas-guided integration of chromatin and transcriptional data, using TF regulatory network inference, identifies region- and condition-specific regulatory states and highlights the hypothalamus as a region that specifically exhibits pronounced condition-dependent TF activity patterns in this dataset.

### 2.2 Perinatal morphine induces hypothalamic chromatin and transcriptomic remodeling that persists to adulthood

**Figure 2:**
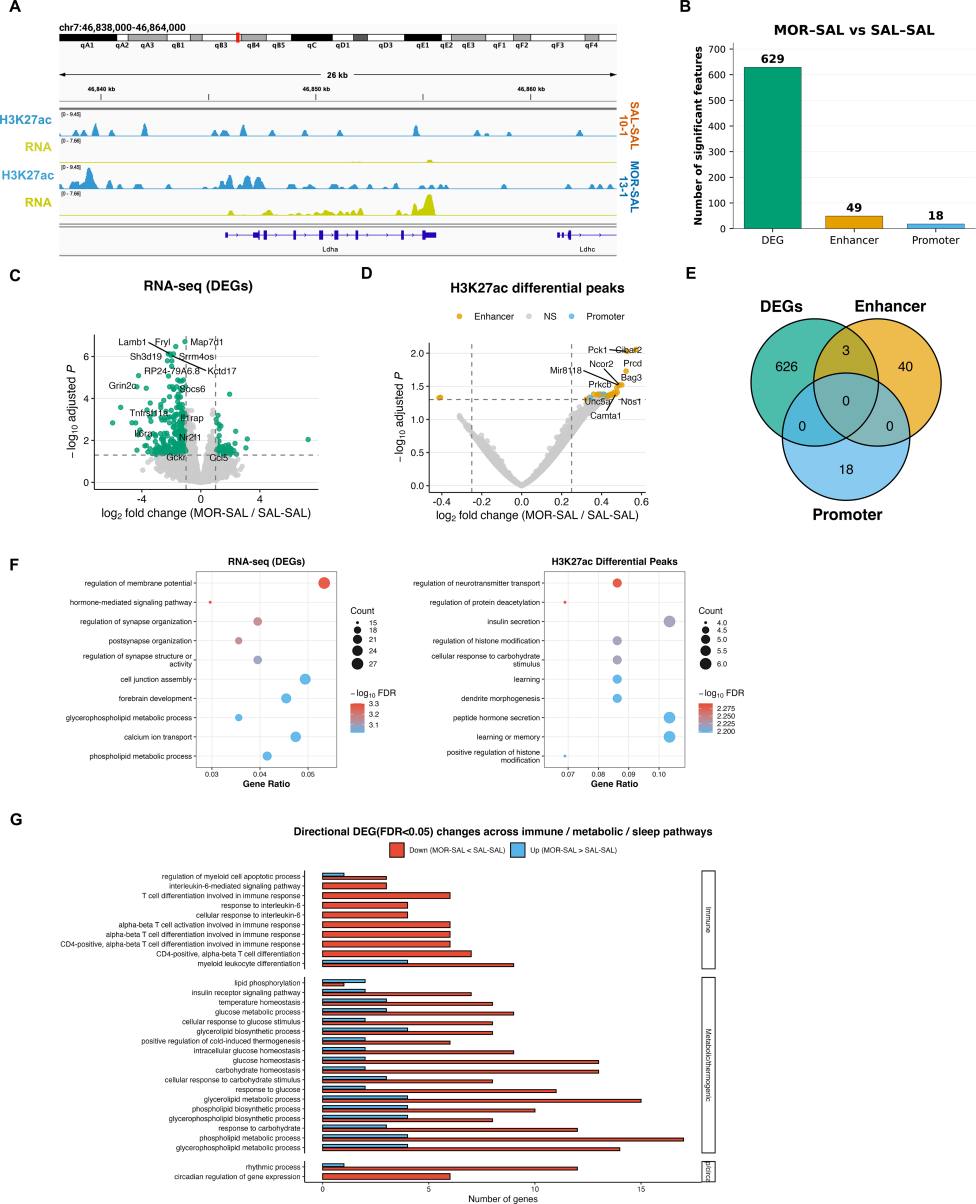
Perinatal morphine exposure alters hypothalamic gene expression programs with limited chromatin changes. **(A)** Representative browser tracks showing H3K27ac and RNA-seq signals in SAL–SAL and MOR–SAL hypothalamus. **(B)** Summary of significantly altered features (FDR < 0.05 and |log₂FC| > 0.25), including enhancers, promoters, and DEGs. **(C)** Volcano plot of differentially expressed genes (SAL–SAL vs MOR–SAL). **(D)** Volcano plot of differential H3K27ac peaks (SAL–SAL vs MOR–SAL), colored by enhancer and promoter annotations. **(E)** Venn diagram showing minimal overlap among genes linked to differential enhancers, promoters, and DEGs. **(F)** Functional enrichment of transcriptional and chromatin alterations in the hypothalamus. **(G)** Directional DEG changes (FDR < 0.05) across immune, metabolic/thermogenic, and sleep/circadian pathways, showing the number of upregulated (blue) and downregulated (red) genes in MOR–SAL relative to SAL–SAL hypothalamus.

Perinatal morphine exposure induced robust transcriptional remodeling in adult hypothalamus, accompanied by comparatively modest, locus-specific changes in H3K27ac. Genome-browser visualizations revealed locus-specific, concordant changes in enhancer-associated H3K27ac signal and RNA abundance at selected metabolic and neuronal loci in MOR–SAL animals relative to SAL–SAL controls. For example, at the *Ldha* locus (lactate dehydrogenase A), MOR–SAL samples show increased H3K27ac signal at the promoter and adjacent regulatory regions, accompanied by increased RNA signal relative to SAL–SAL (**Figure 2A**). At the genome−wide level, MOR–SAL mice exhibited 49 differentially acetylated enhancers and 18 differentially acetylated promoters, compared with 629 differentially expressed genes (DEGs; FDR < 0.05), indicating that perinatal morphine induces a broad transcriptional shift that is accompanied by limited chromatin remodeling (**Figures 2B–D**). The normalized global H3K27ac signal was comparable across all four treatment groups, indicating that group differences in acetylation reflect biological reprogramming rather than technical variation in ChIP quality. (**Supplementary Figure 2E**).

Integration of chromatin and transcriptional outputs further revealed limited one−to−one correspondence between local H3K27ac dynamics and steady−state RNA changes. These data suggest that most transcriptional consequences of perinatal morphine exposure arise through chromatin alterations that are not reflected in differential H3K27ac peak change.

In the hypothalamus, perinatal morphine exposure (MOR–SAL vs SAL–SAL) resulted in robust transcriptional changes but relatively limited H3K27ac remodeling. RNA-seq identified 629 differentially expressed genes (DEGs), but only a small number of differential H3K27ac peaks were observed at enhancers (n = 49) and promoters (n = 18), with no overlap between chromatin-associated genes and DEGs (**Figures 2B–E**). The DEG volcano plot demonstrates marked downregulation of neuronal and metabolic genes, such as *Grin2c*, *Fryl*, and *Lamb1*, along with a smaller subset of upregulated transcripts (**Figure 2C**). Gene ontology analysis of MOR–SAL DEGs revealed coordinated suppression of pathways involved in synaptic organization, postsynaptic signaling, membrane potential regulation, and lipid metabolism, indicating broad transcriptional repression of neuronal and metabolic programs (**Figures 2F–G**). The limited number of H3K27ac differential peaks suggests that the principal transcriptional effects of perinatal morphine exposure in the hypothalamus occur without widespread changes in H3K27ac at annotated enhancers or promoters.

A deeper dissection of the MOR–SAL DEGs revealed that downregulated genes were enriched for small GTPase signaling, postsynaptic organization, and kinase regulation, consistent with the synaptic and signaling deficits observed in the main hypothalamic analysis (**Supplementary Figure 3A**). A distinct subset of DEGs showed downregulation of immune-related pathways, including cytokine-mediated signaling, T-cell activation, and regulation of cytokine production (**Supplementary Figure 3B, D**).

Beyond immune programs, early-life morphine also altered mRNA levels of genes involved in metabolic pathways in the adult hypothalamus. MOR–SAL animals showed coordinated downregulation of phospholipid biosynthesis, glucose-responsive pathways, carbohydrate metabolism, and thermogenic regulators (**Supplementary Figure 3C**). These metabolic DEGs formed a coherent module, with Z-score–scaled heatmaps revealing consistent repression across biological replicates. Together, these results demonstrate that perinatal morphine induces a multi-layered hypothalamic transcriptional phenotype that encompasses synaptic, immune, and metabolic pathways.

### 2.3 Perinatal morphine-induced transcriptomic remodeling under immune challenge conditions

**Figure 3:**
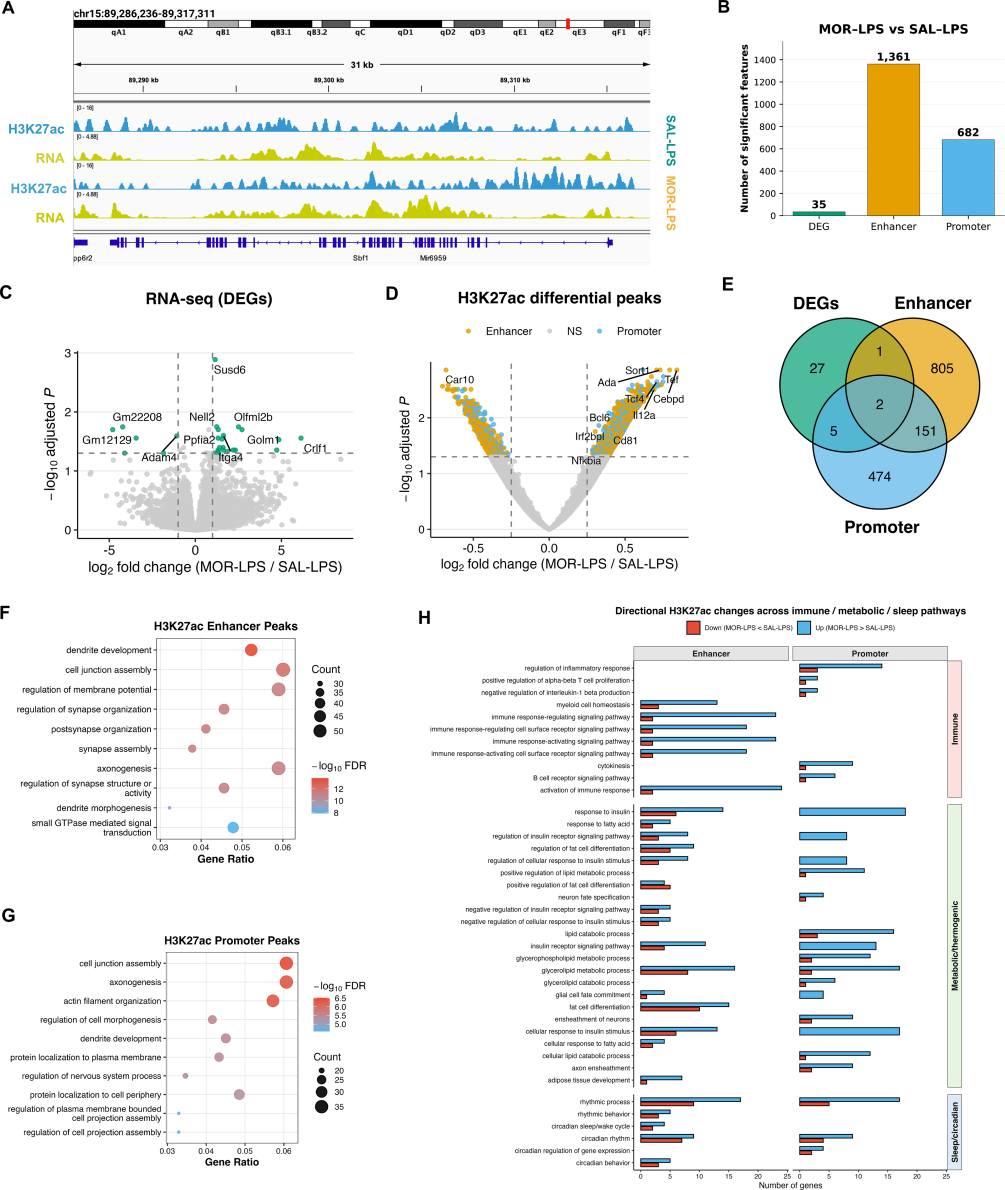
MOR-LPS exposure induces enhancer-dominant chromatin remodeling and metabolic pathway shifts. **(A)** Representative browser tracks showing H3K27ac and RNA-seq signals in SAL–LPS and MOR–LPS conditions. **(B)** Summary of significantly altered features (FDR < 0.05 and |log₂FC| > 0.25), including DEGs, enhancer peaks, and promoter peaks. **(C)** Volcano plot of RNA-seq differential expression (MOR–LPS vs SAL–LPS). **(D)** Volcano plot of differential H3K27ac peaks, separated by enhancer and promoter annotations. **(E)** Venn diagram illustrating overlap among MOR–LPS–responsive DEGs, enhancer-linked genes, and promoter-linked genes. **(F, G)** GO enrichment of differential enhancer and promoter peaks, showing neuronal/synaptic processes at enhancers and membrane-targeting/synaptic localization pathways at promoters. **(H)** Directional Diffpeak changes (FDR < 0.05) across immune, metabolic/thermogenic, and sleep/circadian pathways, showing the number of upregulated (blue) and downregulated (red) genes in MOR–LPS relative to SAL–LPS hypothalamus.

Early exposure to morphine results in significant modification of H3K27ac, particularly at enhancers following acute immunological challenge, while exerting only minor effects on steady-state transcription. Genome browser views at representative loci showed clear exposure-dependent changes in H3K27ac signal, whereas RNA-seq tracks exhibited relatively few alterations, suggesting that enhancer acetylation and immediate mRNA production are not tightly coupled under immune challenged conditions (**Figure 3A**). At the genome-wide level, direct comparison of MOR–LPS and SAL–LPS conditions identified 1361 differential enhancer peaks and 682 differential promoter peaks, but only 35 DEGs, underscoring a marked disparity between chromatin remodeling and transcriptional output (**Figures 3B, C**). Overlap analysis demonstrated negligible concordance among genes linked to altered enhancers, modified promoters, and differentially expressed transcripts, indicating that early-life morphine predominantly alters enhancer-associated regulatory potential during LPS stimulation (**Figure 3E**).

Volcano plot analysis revealed that enhancer-associated peaks exhibited a broad dynamic range of acetylation changes across exposure conditions, whereas promoter-associated peaks showed fewer and more constrained modifications (**Figure 3D**). Functional enrichment analysis of differential enhancer peaks revealed significant associations with axonogenesis, small GTPase signaling, synaptic architecture, and signal transduction, while promoter-associated peaks were enriched for axonogenesis, actin filament organization, and cell junction assembly (**Figures 3F, G**). Directionality analysis across immune, metabolic, and circadian pathways further illustrated divergent patterns of H3K27ac remodeling at enhancers compared with promoters, with immune-related pathways exhibiting particularly pronounced enhancer-level alterations under MOR–LPS relative to SAL–LPS (**Figure 3H**). Collectively, these findings demonstrate that an acute immune challenge produces extensive enhancer-centric chromatin remodeling that is not proportionally reflected in transcriptional output.

Of the 13,788 consensus DiffBind peaks (MOR–LPS vs. SAL–LPS), 2,043 reached FDR<0.05 and |log₂FC| > 0.25 and identified immune-relevant targets including NF-κB pathway loci (*Nfkbia*, *Il12a*), an IRF-family regulator (*Irf2bp*l), and immune co-regulators (Bcl6, Cd81). Notably, *Nfkbia* encodes IκBα, the primary negative regulator of NF-κB, and modest hyperacetylation of the *Nfkbia* promoter (log_2_FC = +0.34, FDR = 0.039) suggests modulation of inhibitory tone rather than classical NF-κB activation. Consistent with this interpretation, several canonical inflammatory and interferon-response loci—including Nfkb1, Il6, Irf7, Ifit1, Mx1, and Oas1a—lacked detectable significant differential H3K27ac peaks in NeuN+ chromatin, consistent with the predominantly non-inflammatory baseline chromatin state of hypothalamic neurons and the absence of classical interferon-stimulated gene induction among the 35 MOR–LPS DEGs. The 67 significant differential peaks in MOR–SAL vs. SAL–SAL annotated primarily to neural and metabolic loci (Pck1, Nos1, Ncor2, Bag3), consistent with the synaptic and metabolic character of the MOR–SAL transcriptomic signature.

Next, we used condition-based contrasts to examine the relationship between baseline chromatin state and stimulus-induced H3K27ac alterations to determine how early-life morphine exposure affects enhancer responsiveness to inflammatory stimulation. For each H3K27ac peak, three complementary comparisons were quantified: (i) baseline differences between exposure conditions prior to immune challenge (MOR–SAL vs SAL–SAL); (ii) the canonical LPS response in saline-reared animals (SAL–LPS vs SAL–SAL); and (iii) LPS-induced responses following early-life morphine exposure, evaluated both within condition (MOR–LPS vs MOR–SAL) and across conditions under identical immune challenge (MOR–LPS vs SAL–LPS) (**Supplementary Figures 4A, B**).

In saline-reared animals, enhancers with slight baseline hyperacetylation in the morphine-exposed group tended to show stronger induction when challenged with LPS. This led to a positive correlation between baseline H3K27ac differences and the normal LPS response, in accordance with the idea that higher basal acetylation indicates a greater response to a stimulus (**Supplementary Figure 4A**). Conversely, in the morphine-exposed condition, baseline H3K27ac levels were negatively correlated with LPS-induced changes. Enhancers with heightened baseline acetylation showed diminished or reversed responses, while hypoacetylated regions exhibited exaggerated induction (**Supplementary Figure 4B**). A comparable inverse correlation was detected when analyzing LPS responses across exposure circumstances under uniform immune stimulation (**Supplementary Figure 4C**).

A direct assessment of LPS-induced H3K27ac alterations across exposure conditions demonstrated a global redistribution of enhancer engagement. Enhancers that were significantly activated by LPS in saline-reared mice generally displayed diminished or negative responses after early-life morphine treatment, whereas sites that were weakly responsive under control circumstances frequently became more inducible (**Supplementary Figure 4D**). Quadrant-based classification of LPS-responsive enhancers indicated that the predominant morphine-associated alterations were response inversions (the direction of enhancer activity is reversed compared to the expected or baseline response), while traditional hyper-activation or hyper-suppression occurred in minor subgroups (**Supplementary Figures 4E, F**).

To delineate the biological programs linked to these morphine-sensitive enhancer classes, GREAT (Genomic Regions Enrichment of Annotations Tool) was utilized on enhancer sets characterized by baseline differences, differential acetylation in response to LPS challenge, and modified LPS responsiveness. Baseline-differential enhancers were predominantly enriched for neural and metabolic pathways, including synaptic architecture, small GTPase signaling, and lipid metabolism. Enhancer sets defined by changes in LPS responsiveness, such as response-inverted and hyper-responsive classes, were more likely to be involved in immune and stimulus-response pathways, including cytokine and chemokine signaling and the regulation of leukocyte-associated processes (**Supplementary Figure 4G**).

Together, these studies demonstrate that early-life morphine exposure modifies inflammatory regulation by altering the correlation between baseline chromatin state and stimulus-induced enhancer activation. Instead of globally inhibiting immune-related regulation, early-life morphine exposure reallocates enhancer involvement within neuronal and immune regulatory networks, leading to a reorganization of immune-responsive regulatory structures after acute inflammatory challenges.

### 2.4 State-dependent rewiring of hypothalamic transcription factor networks following perinatal morphine exposure

Taiji integrates H3K27ac-defined enhancers and RNA-seq expression to construct weighted TF–gene regulatory networks, quantifying TF regulatory influence as a personalized PageRank centrality score (see Methods for full details). GS-TFs were identified by condition-enriched PageRank activity relative to all other conditions.

**Figure 4:**
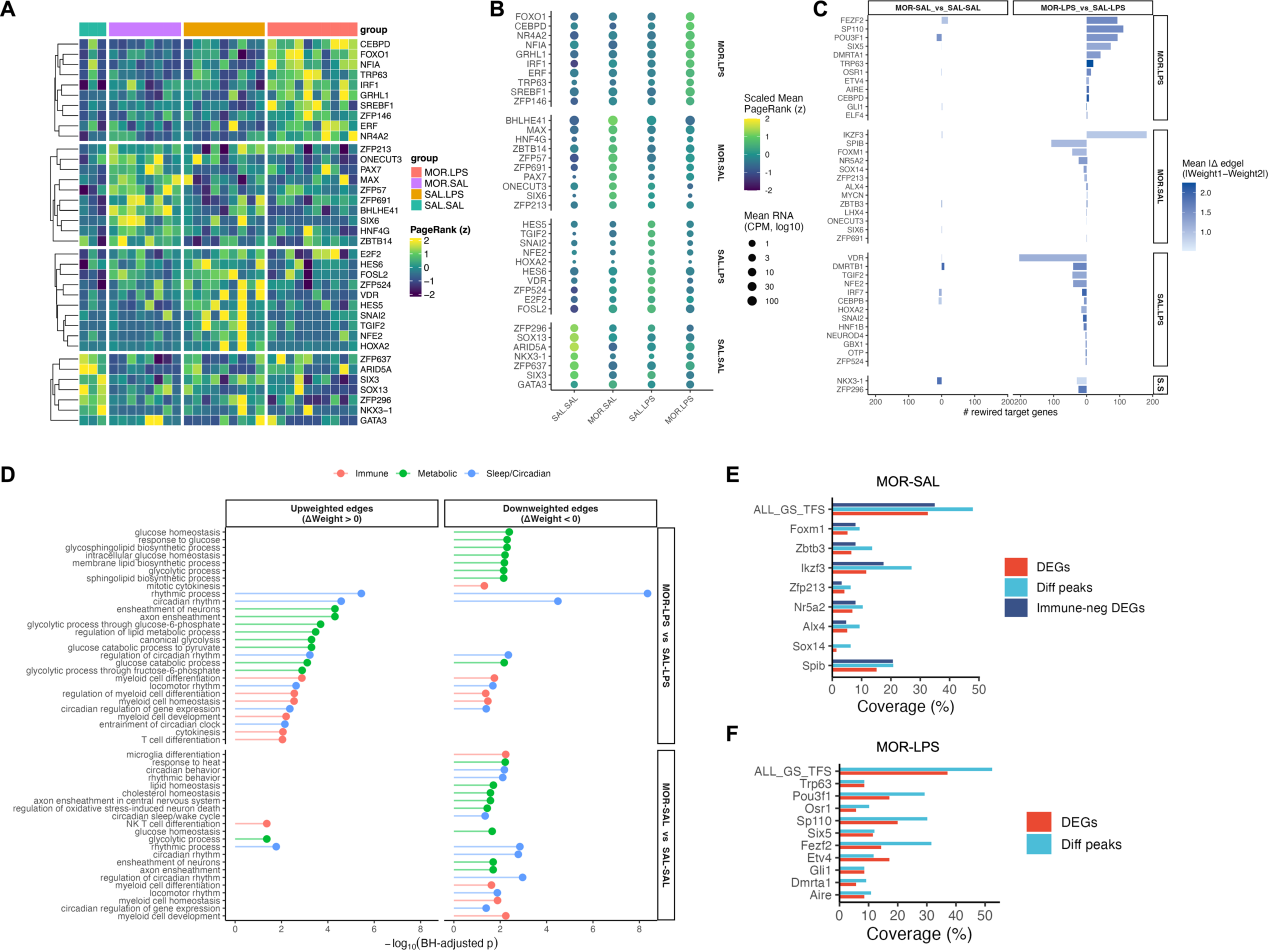
Morphine exposure alters transcription factor regulatory network architecture. **(A)** Heatmap of Taiji transcription factor (TF) activity scores (PageRank z-scores) across four conditions (SAL–SAL, MOR–SAL, SAL–LPS, MOR–LPS). TFs are hierarchically clustered by PageRank profiles; colors indicate scaled TF centrality within condition-specific regulatory networks. **(B)** Summary of TF activity across conditions. Dot color denotes scaled mean PageRank (z-score), and dot size reflects mean RNA expression (CPM, log_10_). TFs are grouped by condition-specific TF sets. **(C)** Network rewiring summary for MOR–SAL vs SAL–SAL and MOR–LPS vs SAL–LPS. Diverging bars show the number of rewired target genes per TF; bar color represents the mean absolute change in edge weight (|Weight_1_ − Weight_2_|). **(D)** Gene Ontology enrichment of rewired target genes. Lollipop plots display enriched immune, metabolic, and sleep/circadian processes among targets of upweighted (ΔWeight > 0) and downweighted (ΔWeight < 0) edges. The x-axis indicates −log_10_(BH-adjusted p value). **(E, F)** Coverage of condition-specific TF target genes in MOR–SAL **(e)** and MOR–LPS **(f)**. Bars indicate the percentage overlap with differentially expressed genes (DEGs), differential peaks, or immune-negative DEGs. “ALL_GS_TFS” denotes the union of group-specific TFs.

Perinatal morphine exposure results in condition-specific reorganization of hypothalamic transcription factor (TF) networks (**Figures 4A–F**). To systematically characterize regulatory changes associated with morphine-induced transcriptional and chromatin alterations, we employed Taiji to infer TF–target networks by integrating H3K27ac profiles with RNA expression across all experimental groups. This approach enables simultaneous quantification of TF PageRank centrality, TF expression, and weighted TF–target regulatory edges, offering a network-level perspective of condition-dependent regulatory architecture.

Taiji analysis identified largely distinct sets of group-specific transcription factors (GS-TFs) across SAL–SAL, MOR–SAL, SAL–LPS, and MOR–LPS conditions (**Figure 4A**). GS-TFs were defined by condition-specific PageRank z-scores and RNA expression specificity within a shared TF–target backbone. Visualization of PageRank z-scores (**Figure 4A**) and integration with TF RNA abundance (**Figure 4B**) demonstrated substantial reorganization of TF hierarchy in morphine-exposed animals. SAL–SAL samples displayed relatively uniform TF centrality with minimal specialization, whereas MOR–SAL and MOR–LPS conditions exhibited pronounced shifts in both TF ranking and expression. Baseline MOR–SAL GS-TFs included Foxm1, Ikzf3, and Spib, while MOR–LPS GS-TFs included Fezf2, Trp63, and Sp110, indicating recruitment of distinct TF sets at baseline and following immune challenge.

To quantify changes in regulatory wiring, TF–target edges were compared between MOR–SAL vs SAL–SAL and MOR–LPS vs SAL–LPS using Wilcoxon rank-sum tests on Taiji edge percentile ranks. Edges were classified as significantly rewired if they exceeded both an absolute change threshold (|Δ weight| > 0.5) and statistical significance (P < 0.05). For each GS-TF, a rewiring index was calculated as the mean absolute change in edge weight across all significantly rewired targets, together with the total number of rewired edges. Ranking by these metrics identified TFs exhibiting the largest shifts in regulatory connectivity between conditions (**Figure 4C**). In MOR–SAL relative to SAL–SAL, Foxm1, Ikzf3, and Spib showed the highest rewiring indices. In MOR–LPS relative to SAL–LPS, Sp110, Fezf2, and Trp63 exhibited the strongest changes. This indicates condition-dependent redistribution of TF regulatory influence.

Functional annotation of rewired targets was conducted using Gene Ontology (GO) biological process enrichment analysis on pooled target sets, stratified by the direction of edge weight change (upweighted or downweighted) and by comparison (**Figure 4D**). GO terms were categorized into immune, metabolic, and sleep/circadian groups based on keyword matching. In MOR–LPS compared to SAL–LPS, upweighted edges were enriched for metabolic and neuronal processes, including lipid and glucose metabolism, neuronal differentiation, and synaptic organization. Downweighted edges were enriched in immune-related pathways, including cytokine signaling, interferon response, and leukocyte activation. In MOR–SAL compared to SAL–SAL, rewired targets were primarily associated with immune and inflammatory processes, consistent with altered baseline regulatory connectivity in the absence of acute immune stimulation.

To assess the extent to which GS-TF networks capture observed epigenomic and transcriptional differences, we quantified the proportion of differential H3K27ac peaks and DEGs linked to GS-TF–target edges with high Taiji weights (>2). In MOR–LPS, individual GS-TFs, such as Sp110, Fezf2, and Trp63, were associated with substantial fractions of both differential H3K27ac peaks and DEGs (**Figure 4F**), indicating a broad regulatory reach under immune challenge. In the MOR–SAL condition, GS-TFs overlapped with both DEGs and differential H3K27ac peaks, including DEGs outside immune-related gene sets (**Figure 4E**). In contrast, the MOR–LPS versus SAL–LPS comparison showed limited numbers of DEGs despite widespread H3K27ac differences (**Figure 4F**).

High-confidence, condition-specific TF subnetworks were extracted from the global TF–target backbone by applying stringent filtering criteria (edge weight above the 98th percentile, absolute Pearson correlation greater than 0.3, and retention of the top 25 targets per TF). The resulting subnetworks demonstrate that GS-TFs are integrated within a shared enhancer framework but form distinct, state-dependent modules. MOR–SAL subnetworks were enriched for immune-associated TFs and targets, while MOR–LPS subnetworks emphasized targets related to neuronal, metabolic, and stress-associated processes. Collectively, these analyses demonstrate that perinatal morphine exposure leads to state-dependent reorganization of hypothalamic TF network architecture at baseline and following immune challenge.

Genome-wide edge-weight comparisons identified 1,380 significantly rewired edges in MOR–SAL vs. SAL–SAL, versus 10,884 in MOR–LPS vs. SAL–LPS—an approximately eight-fold expansion under immune challenge (**Supplementary Figures 6B, C**). MOR–SAL showed a net loss of connectivity (474 gaining vs. 906 losing edges), consistent with morphine-induced regulatory pruning at baseline; MOR–LPS showed a net gain (7,145 gaining vs. 3,739 losing edges), reflecting large-scale network expansion when immune challenge is superimposed on developmental morphine exposure.

This asymmetry mirrors the broader epigenomic pattern: baseline morphine imprints a focused, pruned regulatory state, while the combination of perinatal morphine exposure and acute immune challenge in adulthood drives a substantially amplified network reorganization.

To identify genes with convergent epigenomic and network evidence, DEGs and DiffBind peaks at FDR < 0.05 were intersected with significantly rewired Taiji edges. In MOR–LPS, eight genes showed both significant differential expression and differential H3K27ac: TCF4/E2-2, MGLL, SORT1, SLC27A1, SBF1, OLFML2B, PSD3, and ABR. Seven showed concordant directionality (upregulated RNA with hyperacetylated H3K27ac regulatory elements); ABR was discordant, with upregulated RNA (log2FC = +0.54) but hypoacetylated associated peaks, suggesting post-transcriptional or indirect mechanisms regulate expression. Among concordant genes, TCF4/E2-2 (also known as E2–2 or ITF2; encoded by Tcf4) exhibited the strongest multi-layer signature: upregulation in MOR–LPS DEGs (log2FC = +1.32, padj = 0.044), association with seven hyperacetylated regulatory elements (DiffBind FDR < 0.05), and receipt of 23 significantly rewired upstream TF–target edges, 22 of which gained weight in MOR–LPS. Because TCF4/E2–2 is a neuronal basic helix-loop-helix transcription factor implicated in neurodevelopmental and synaptic gene regulation, its convergence across RNA expression, chromatin acetylation, and TF-network layers nominates it as a biologically plausible regulator of the MOR–LPS neuronal state. In the MOR–SAL comparison, four genes satisfied the DEG ∩ DiffPeak criterion (BLCAP, LDHA, PLEKHD1, SDK2), none of which had Wilcoxon-significant rewired edges, consistent with more limited chromatin–transcription–network coupling at baseline. These results identify TCF4/E2–2 as the single gene at which all three regulatory layers converge in MOR–LPS, providing a prioritized candidate for mechanistic follow-up studies (**Supplementary Figure 6E**).

### 2.5 Cytokine levels are altered in adulthood following perinatal opioid exposure

**Figure 5:**
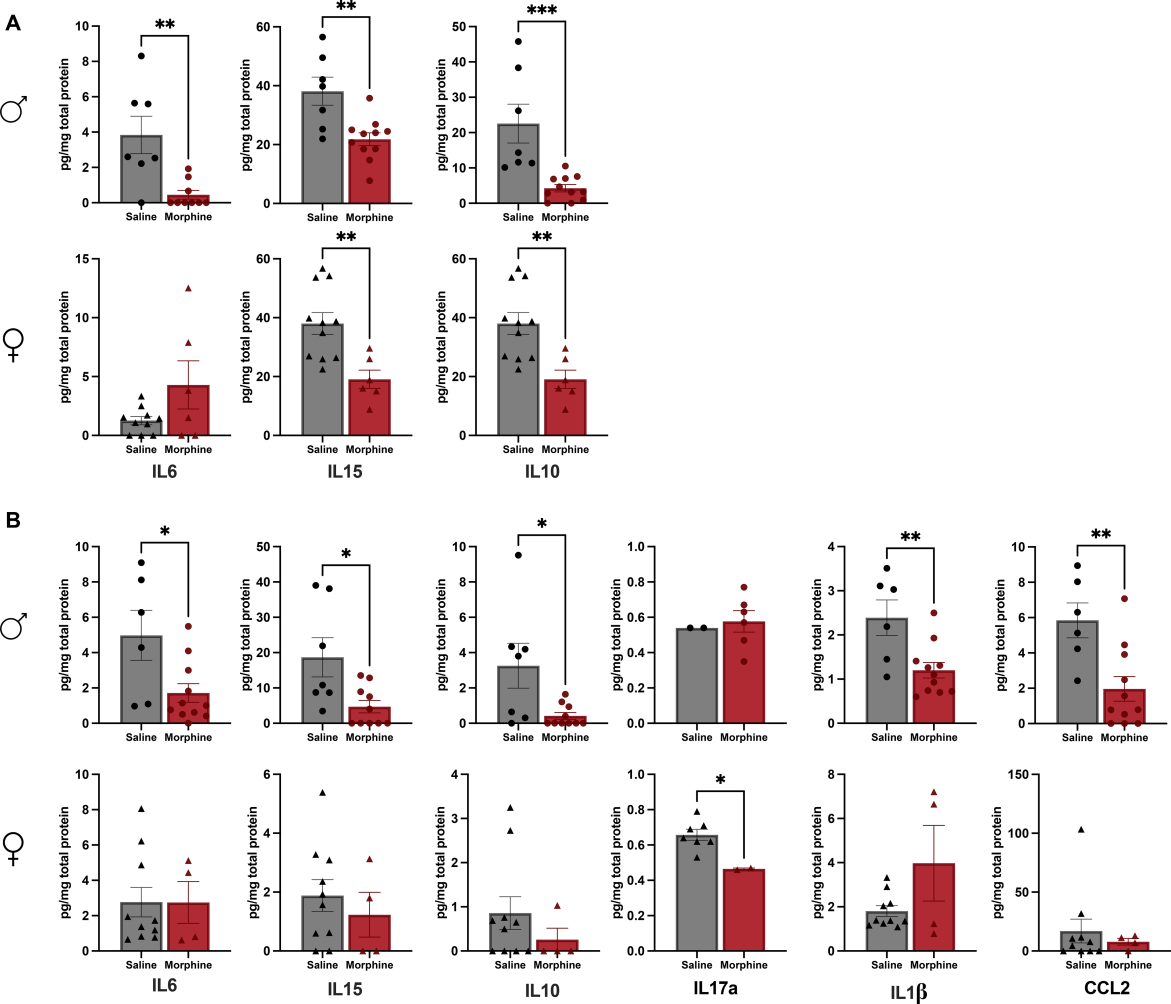
Cytokine levels are altered by perinatal morphine exposure in adulthood. Graphs showing levels of cytokines altered by perinatal morphine exposure in adult males and females. **(A)** Levels of selected cytokines in the hypothalamus in males (upper) and females (lower) **(B)** Levels of selected cytokines in the spleen in males (upper) and females (lower) *p< 0.05; **p< 0.01, ***p<0.001.

Because enhancers drive downstream gene expression, inversion-dominated regulation could reverse or reshape entire transcriptional programs in cells exposed to both LPS and morphine, affecting inflammation and downstream phenotypes. Therefore, we sought to determine whether such reprogramming is manifested at the protein level. We focused on several cytokines known to be altered by opioids, as no specific cytokines were directly highlighted in our sequencing analyses. Cytokine levels were measured in the hypothalamus and spleen to assess both neuroimmune and systemic immune function. Changes in cytokine levels were minimal at PND14. Morphine-treated (MOR) males showed increased levels of IL2 in the hypothalamus and decreased levels of IL1β in the spleen (**Supplementary Figure 7**). MOR females displayed decreased levels of IL15 and IL10 in the hypothalamus and IL6 and IL12p70 in the spleen, and increased levels of IL1β and IL4 in the spleen (**Supplementary Figures 7**). No changes in cytokine levels were observed at PND15 in any tissue in either sex (**Supplementary Figure 7**). In striking contrast, adult MOR males had decreased levels of IL15, IL6, and IL10 in the hypothalamus (**Figure 5A**) and decreased levels of IL15, IL1β, IL6, IL10, and CCL2 in the spleen (**Figure 5B**). Adult MOR females had decreased levels of IL15 and IL10 in the hypothalamus (**Figure 5A**) and decreased levels of IL17a in the spleen (**Figure 5B**). For the complete summary of all cytokines measured, see **Supplementary Tables 2**, **3**.

### 2.6 Perinatal morphine exposure blunts response to adult immune challenge in males

**Figure 6:**
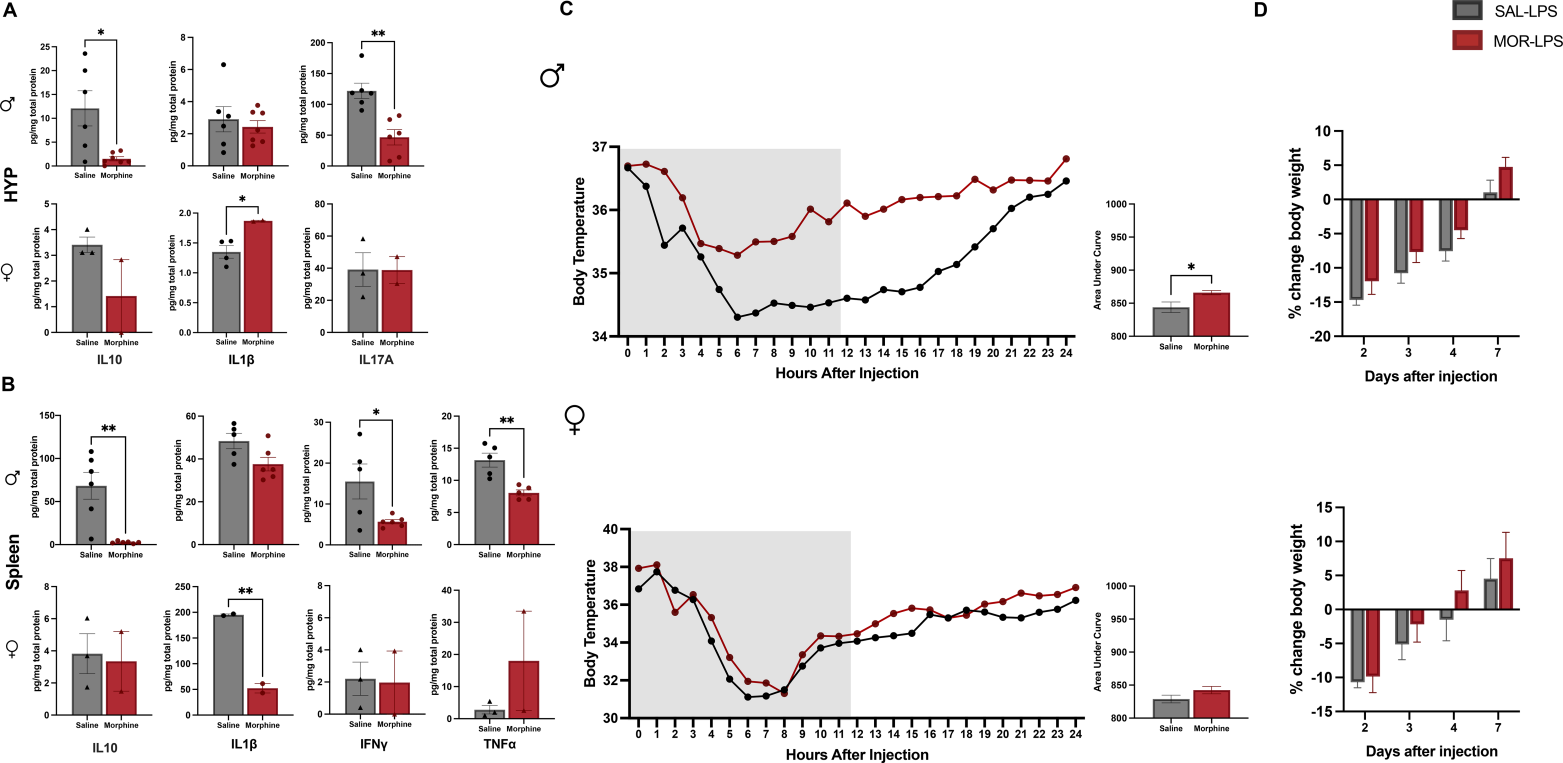
Perinatal morphine exposure blunts response to adult immune challenge. **(A)** Graphs showing cytokine levels 2 hours following injection with LPS in the hypothalamus in males (upper) and females (lower) **(B)** Graphs showing cytokine levels 2 hours following injection with LPS in the spleen in males (upper) and females (lower) **(C)** Core body temperature measurements in the 24 hours following LPS administration in males (upper) and females (lower), calculated area under the curve shown in corresponding bar graph **(D)** Percent change in body weight measured over a week following LPS administration in males (upper) and females (lower) For all graphs, saline bars shown in gray and morphine bars shown in red. Circles= males and triangles= females. *p< 0.05; **p< 0.01.

Next, we sought to investigate whether immune alterations resulting from perinatal opioid exposure impact the systemic response to a future immune challenge in adulthood. Adult mice were injected with LPS, and cytokines were measured in the hypothalamus and spleen 2 hours later to assess the acute inflammatory response. Male MOR-LPS mice showed reductions in levels of IL10 and IL17a in the hypothalamus (**Figure 6A**) and decreased levels of IL10, IFNγ, and TNFα in the spleen (**Figure 6B**) compared to controls. Female MOR-LPS mice had increased IL1β in the hypothalamus (**Figure 6A**) and decreased IL1β in the spleen (**Figure 6B**). For the complete summary of all cytokines measured, see **Supplementary Tables 2** and **3**. We also measured physiological responses to the LPS challenge. This high dose of LPS induces hypothermia in mice, but this effect is significantly reduced in MOR-LPS males compared to the SAL-LPS controls (**Figure 6C**). MOR-LPS males also did not display as drastic LPS-induced weight loss as SAL-LPS mice, though this difference did not reach significance (**Figure 6D**). In contrast, female MOR-LPS mice did not show differences in body temperature or LPS-induced weight loss relative to SAL-LPS females (**Figures 6C, D**).

### 2.7 Perinatal morphine exposure alters physiological properties in adult males

**Figure 7:**
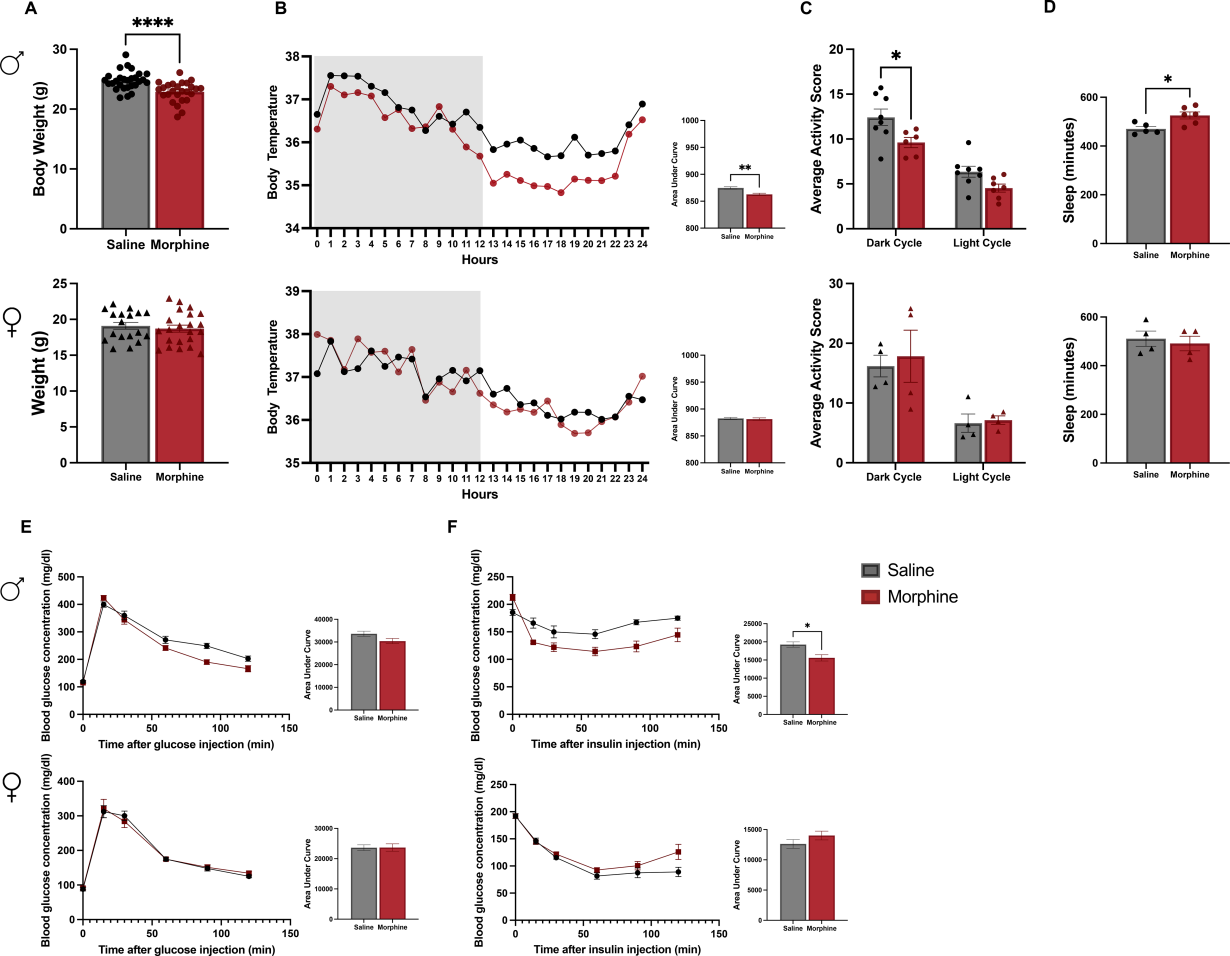
Perinatal morphine exposure alters physiology and insulin response in adult males **(A)** Morphine males weighed less than saline controls in adulthood (upper), though morphine females did not show a significant difference in body weight (lower) **(B)** Both morphine males had decreased core body temperature throughout the light cycle (upper), but morphine females did not (lower). Calculated area under the curve shown in corresponding bar graph **(C)** Morphine males showed a reduction in average activity score in the dark cycle (upper), but females did not display any differences in activity level (lower) **(D)** Morphine males showed an increase in total sleep time (upper), but morphine females did not show any changes in sleep (lower) **(E)** Blood glucose concentrations following glucose injection in GTT in males (upper) and females (lower) **(F)** Blood glucose concentrations following insulin injection in ITT in males (upper) and females (lower) For all graphs, saline bars shown in gray and morphine bars shown in red. Circles= males and triangles= females. *p< 0.05; **p< 0.01.

Several baseline-differential enhancers were predominantly enriched for neural and metabolic pathways when comparing morphine (MOR) and saline (SAL) conditions, prompting us to investigate persisting physiological effects of perinatal morphine exposure. We measured several physiological characteristics, including changes in body weight, body temperature, activity, and sleep. MOR males weigh less than SAL controls in adulthood and display an overall reduction in basal core body temperature (**Figures 7A, B**). MOR females do not have a significant difference in body weight or core body temperature (**Figures 7A, B**). MOR males show a reduction in average activity score in the dark cycle and increased total sleep time (**Figures 7C, D**), but MOR females do not show any changes in activity or sleep (**Figures 7C, D**).

### 2.8 Perinatal morphine exposure alters glucose response

Multiple H3K27ac promoter peaks identified in our sequencing studies were located at genes involved in glucose metabolism (**Figure 2**; **Supplementary Figure 4**). To investigate if these transcriptomic changes translated into broader physiological responses, we conducted glucose tolerance test (GTT) and insulin tolerance test (ITT). Male MOR mice do not differ from controls in the GTT (**Figure 7E**) but have significantly lower blood-glucose concentrations during the ITT, indicating an enhanced response to insulin (**Figure 7F**). MOR females do not differ from controls in the GTT or the ITT (**Figure 7**).

## 3 Discussion

Infants exposed to opioids *in utero* are at high risk for developing Neonatal Opioid Withdrawal Syndrome (NOWS). However, environmental factors and challenges in conducting long-term clinical studies have hindered the identification of lasting effects. While rodent models can mimic acute withdrawal and developmental delays observed in human NOWS, consistent long-term phenotypes are rarely reproduced, with many studies showing no enduring effects altogether (9). These inconsistencies have made it difficult to elucidate long-term phenotypes. Further, it is possible that there are latent phenotypes induced by perinatal opioid exposure that only arise once a secondary stressor has been introduced. A small number of studies have begun to investigate these potential effects, including outcomes arising from future traumatic brain injury (11) or adolescent nicotine exposure (10). Thus, in the present study, we sought to employ a high-level, hypothesis-generating screening approach to identify potential biological systems that are affected by perinatal morphine exposure directly or that may be rendered vulnerable to other stimuli following opioid exposure. As detailed above, we discovered significant epigenetic and transcriptomic changes in immune and metabolic pathways, and prioritized studying phenotypes associated with these systems.

Opioids are immunomodulators, acting both systemically and within the central nervous system (19). There is evidence that perinatal opioid exposure can alter the immune system and cause upregulation of microglia and a release of pro-inflammatory cytokines within the central nervous system (20–24). Clinical studies corroborate these data, showing that infants exposed to opioids *in utero* have increased rates of infection and hospitalization (25). Still, the extent of this immune dysfunction, as well as the molecular mechanisms underlying the relationship between perinatal exposure and the immune system, have yet to be fully characterized.

In the present study, we identify significant downregulation of immune-related pathways, including cytokine-mediated signaling. Further, perinatal morphine exposure alters the transcriptional landscape such that there are distinct alterations in responses to an immune challenge, specifically within chromatin remodeling. Taken together, our data indicates that early-life opioid exposure installs a stable baseline H3K27ac imprint at hypothalamic enhancers, selectively attenuates synaptic and metabolic transcriptional programs, and suppresses basal immune gene expression. This reconfigured landscape has broader consequences, as it significantly alters the transcriptional response to an immune challenge by LPS in adulthood. This framework supports a model in which perinatal morphine lowers basal hypothalamic immune function while reconfiguring the enhancer architecture that interprets inflammatory stimuli, leading to redistributed, and often inverted, patterns of LPS-induced activity across synaptic and immune regulatory networks. Within these studies, multiple mice per litter were treated as individual variables. We acknowledge that utilizing this approach without variance correction for litter effects is a potential limitation of our bioinformatic approach, and that some identified target genes could represent litter-driven effects. However, animals were distributed across 36 litters (8–11 per condition), and the consistency of effects across multiple immune and metabolic pathways provides confidence in the biological relevance of our findings.

The enrichment of GO pathways and TFs associated with immune function prompted us to quantify cytokine levels to better understand how these transcriptional changes manifest at higher levels. We measured cytokine levels at PND14 (while mice are in an opioid dependent state) and at PND15 (during spontaneous opioid withdrawal), and in adulthood. Though alterations in cytokine levels are mild at PND14, the few changes present, primarily the increase in proinflammatory IL2 in males and decrease in anti-inflammatory IL10 in females, are indicative of early life hyperinflammation (**Supplementary Figure 7**). At PND15, there are no changes in cytokines in any tissue, indicating that the withdrawal period may normalize the changes initially induced by perinatal morphine exposure. This may be due to the involvement and recruitment of glia during neonatal opioid withdrawal (26), which can further alter cytokine levels (27, 28), and, thus, would effectively reverse any initial changes induced by morphine alone.

The relatively mild alterations observed at PND14/15 are in stark contrast to the large number of cytokines that are suppressed in adulthood, suggesting that early-life epigenetic effects become augmented throughout development. Levels of multiple cytokines are also suppressed following LPS administration. These changes are particularly striking in the spleen, indicating that the peripheral immune system may be more vulnerable to the effects of perinatal morphine. Across multiple tissues and timepoints, levels of IL10 and IL1β are consistently altered by morphine. These specific cytokines can be used to guide more targeted studies into the immunomodulatory effects of perinatal morphine.

The immunosuppression observed at the transcriptional and molecular levels further manifests as alterations in physiological response to an immune challenge. Morphine-exposed male mice do not display as severe of a hypothermic response that would be typical of the high dose of LPS used in this study (29), nor do they exhibit as drastic LPS-induced weight loss in the period following injection. We hypothesize that the early-life inflammation induced by perinatal morphine leads to a compensatory, immunosuppressed phenotype in adulthood. Other studies have shown that a neonatal immune challenge can induce lasting alterations to innate immunity, culminating in an attenuated immune response in adulthood (30–33). This study is the first to characterize the impact of perinatal opioid exposure on immune function, identifying alterations at the transcriptomic, molecular, and physiologic levels, in both early-life and adulthood, and under basal and immune-challenged conditions— all within the same opioid exposure paradigm. Taken together, these data indicate that perinatal morphine suppresses baseline immune function, which can impact future responding to an immune challenge.

Opioids can affect several physiologic properties, including weight, sleep, thermoregulation, glucose metabolism, and hormone release, which may, in part, contribute to metabolic disorder in patients with opioid use disorder (34–36). Impaired physiologic development following perinatal exposure has been well established in both clinical studies and in animal models in neonates (9, 37, 38), but more specific effects of opioid-induced metabolic dysfunction in neonates, as well as potential persisting effects, have yet to be established (39). Our sequencing studies identified alterations in metabolic pathways, prompting further investigation into potential dysfunction of this system. Gene ontology analysis of MOR–SAL DEGs demonstrated coordinated downregulation of pathways involved in synaptic organization, postsynaptic signaling, and phospholipid/glycerophospholipid metabolism, indicating broad suppression of synaptic and metabolic gene programs. Physical measurements corroborate these findings, as male mice exposed to morphine in the perinatal period have lower body weights in adulthood. These mice also display lower core body temperatures, have decreased activity, and increased sleep. The improved insulin sensitivity, shown in **Figure 7F**, is likely the consequence of reduced weight in these mice.

Guided by our transcriptomic and epigenomic studies, we utilized molecular assays and assessed physiologic changes to further explore the effects of perinatal opioid exposure on immune functioning and metabolism. Mice were examined both under basal conditions and following an immune challenge, enabling interrogation of morphine’s enduring effects on both resting-state and in response to an inflammatory ‘secondary stressor’. This integrative strategy allows for the identification of enhancer remodeling, transcriptional reprogramming, and transcription factor network alterations to better understand how early-life opioid exposure reshapes immune and metabolic development and produces lasting physiological consequences.

## 4 Materials and methods

### 4.1 Animals

Male and female C57Bl/6NTac mice (referred to as B6 throughout; Taconic, Hudson, New York) were bred at the University of Pennsylvania. Upon weaning, mice were group-housed (3-5/cage) with ad libitum access to food and water under a 12-h light/dark cycle (lights on at 0700). All procedures were approved by the University of Pennsylvania Animal Care and Use Committee and were in compliance with the National Institutes of Health Guide for the Care and Use of Laboratory Animals. Animals were distributed across 36 distinct litters (SAL-SAL: 8, MOR-SAL: 11, SAL-LPS: 8, MOR-LPS: 9); all litters contained more than one offspring. Perinatal treatment was assigned at the dam level; adult immune challenge was assigned at the individual animal level after weaning. Litter was not included as a model covariate because it is nested within perinatal condition (the MOR and SAL litter pools are entirely non-overlapping in the primary contrasts), making litter collinear with condition. Detailed sample composition and sex ratios are provided in **Supplementary Table 1**. The statistical unit is the individual animal.

### 4.2 Drugs

Morphine sulfate was obtained from NIDA Drug Supply (Research Triangle Park, NC) and dissolved in 0.9% saline.

### 4.3 Perinatal morphine exposure paradigm

Mice were exposed to morphine throughout gestation and for the first two postnatal weeks. This model delivers opioid exposure for the equivalent of the entirety of three trimesters of a human pregnancy and produces developmental delays and spontaneous opioid withdrawal upon cessation of morphine (18). Briefly, 8-12-week-old female mice were implanted with osmotic minipumps (model 1004; Alzet, Cupertino CA) delivering either morphine (10 mg/kg/day) or saline and then co-housed with a drug-naïve male to mate. Within 4–8 hours after birth, pups were transferred to a drug-naïve surrogate dam. Third trimester equivalent exposure began on the evening of postnatal day 1 (PND1) and continued until the morning of PND14. Morphine was delivered at a dose of 10 mg/kg in a volume of 10 mL/kg and administered subcutaneously twice daily at 9:00 AM and 5:00 PM. The last injection of morphine was given on the morning of PND14. Following the final morphine injection, mice underwent spontaneous opioid withdrawal. Control mice received saline in an equal injection volume. A total of 4 distinct cohorts were used for transcriptomic, molecular, and behavior analysis, with each mouse used as an individual sample.

### 4.4 Tissue collection

To study the effects of perinatal morphine on cytokine levels, tissue was collected at distinct timepoints from separate cohorts in the early postnatal period (PND14 and PND15) and in adulthood. The PND14 samples were collected 2 hours following the final morphine injection to capture the effects of the morphine-dependent state, while the PND15 samples were collected 24 hours after the final injection, during spontaneous opioid withdrawal. This yielded two experimental groups in the early postnatal stage: saline controls (SAL) and morphine exposed mice (MOR). To study persisting transcriptomic and molecular effects of perinatal opioid exposure, quantification of cytokine levels and RNA-seq/ChIP-seq was performed in tissue collected from a separate cohort of adult mice, assessed both at baseline and in response to an immune challenge. Tissue was collected at 2 hours following an injection of saline or LPS, resulting in four experimental groups for adult measurements: saline controls +/- LPS (SAL-SAL and SAL-LPS) and morphine mice +/- LPS (MOR-SAL and MOR-LPS). PFC, hippocampus, hypothalamus, striatum, and spleen were collected by gross dissection in all mice. Samples were flash-frozen after excision and stored at -80 °C until processed. For overview of experimental groups, see Experimental Timeline, **Supplementary Figure 1**.

### 4.5 Chromatin profiling (MOWChIP-seq)

#### 4.5.1 Nuclei isolation and sorting

Bilateral punches (2.0 mm) were collected from the PFC, hippocampus, and striatum and two adjacent punches from hypothalamus, rapidly frozen, and stored at –80 °C until use. To prevent the potential batch effect, all tissue samples were randomized prior to processing. Nuclei were isolated using a cold extraction buffer, homogenized with a Dounce tissue grinder, filtered (40 µm), and pelleted by centrifugation, followed by iodixanol gradient centrifugation (17, 40, 41). Nuclei were resuspended and immunolabeled with anti-NeuN antibody conjugated to Alexa Fluor 488 (MAB377X, EMD Millipore) at 4 °C for 1 h. NeuN+ fraction was sorted using FACS (BD Biosciences).

#### 4.5.2 MNase digestion and chromatin immunoprecipitation

Approximately 10,000 NeuN+ nuclei from a single animal were used to produce one independent ChIP-seq library per animal per brain region (one biological replicate per library). Nuclei were lysed and digested with MNase (88216, Thermo Fisher) in the presence of CaCl_2_, and the reaction was quenched with EDTA. Fragmented chromatin was collected from the supernatant after centrifugation. For each MOWChIP assay, 5 µL of protein A Dynabeads (10001D, Thermo Fisher) were coupled with 0.125 µg of H3K27ac antibody (39135, Active Motif), and immunoprecipitation was performed using the previously established MOWChIP-seq protocol (15–17). Sex composition of ChIP-seq samples per condition and region is provided in **Supplementary Table 1**.

#### 4.5.3 Library construction and sequencing

Purified ChIP DNA (from ~10,000 nuclei) and input DNA (from ~2,000 nuclei) were used for library construction. Libraries from the PFC were prepared using the xGen™ 2S DNA Library Prep Kit (10009877, IDT), whereas libraries from hippocampus, hypothalamus, and striatum were prepared using the ThruPLEX DNA-Seq kit (R400674, Takara Bio). Library fragment size was checked using High Sensitivity DNA Analysis Kit (5067-4626, Agilent) on a TapeStation System (G2992AA, Agilent), and primer-dimers were removed using SPRIselect beads purification. Libraries were pooled based on the concentrations quantified by a KAPA Library Quantification Kit (4824, Roche). Sequencing was performed on an Illumina NovaSeq X Plus platform (paired-end 150 nt, ~15 million reads/library).

#### 4.5.4 ChIP-seq data processing and differential analysis

Adapters and low-quality bases were trimmed using Trim Galore (v0.6.7). Reads were aligned to the *Mus musculus* mm10 reference genome with Bowtie2 (v2.4.5) (42), and PCR duplicates were removed using SAMtools (v1.15.1) (43). Reads overlapping ENCODE mm10 blacklist regions were excluded with BEDTools subtract (v2.30.0) (44). Genome-wide coverage profiles were generated with bedtools genomecov and converted to BigWig format with bedGraphToBigWig (45). Peak calling was performed with MACS2 (v2.2.7.1; parameters: -q 0.05 -g mm) (46).

Differential peak analysis was performed separately for each brain region. To enable consistent quantification across replicates and conditions, a unified consensus peak set was constructed in DiffBind (v3.10.1) using condition-aware peak merging. Peaks were retained if present in at least 50% of samples across all conditions, and consensus peaks overlapping ENCODE mm10 blacklist regions were removed prior to downstream analysis.

Read counts were quantified over the blacklist-filtered consensus peak set using DiffBind (dba.count) with summarizeOverlaps. Peaks were filtered for robust coverage using a condition-aware criterion: a peak was retained if it contained ≥15 reads in at least 80% of samples within any experimental condition. The filtered peak set was re-quantified and used for all differential analyses. Library-size normalization and dispersion estimation were performed using DESeq2 as implemented in DiffBind.

Differential H3K27ac peaks were identified using DESeq2-based testing via DiffBind (dba.analyze) for prespecified contrasts, including MOR–SAL vs SAL–SAL (baseline exposure effect), MOR–LPS vs SAL–LPS (exposure effect under immune challenge), and within-condition LPS responses where indicated. Unless explicitly stated otherwise, peaks were considered differentially acetylated at FDR < 0.05 (Benjamini–Hochberg). Where indicated, an additional effect-size filter (|log2FC| > 0.25) was applied. Complete differential binding reports were generated for each comparison using dba.report (threshold = 1) and exported as CSV files for downstream annotation. Effect sizes are reported as DESeq2 log2 fold changes. The DiffBind/DESeq2 design formula was ~sex + condition. Litter was not included as a covariate because it is nested within perinatal condition (see §4.1). The statistical unit is the individual animal.

Differential peaks were annotated to genomic features using ChIPseeker (v1.36.0) with gene coordinates from TxDb.Mmusculus.UCSC.mm10.knownGene. Each peak was assigned to the nearest annotated feature (promoter, intron, exon, 3’ UTR, 5’ UTR, or distal intergenic region). Promoter-associated peaks were defined as overlapping the TSS ± 1 kb; all remaining peaks were classified as enhancer-like (non-promoter) H3K27ac peaks. Genes associated with significant peaks were subjected to Gene Ontology (Biological Process) enrichment analysis using clusterProfiler (v4.8.3) with a hypergeometric test; P values were adjusted using the Benjamini–Hochberg method.

### 4.6 RNA-seq

#### 4.6.1 RNA-seq library preparation

Approximately 5,000 NeuN+ nuclei from a single animal were used to produce one independent RNA-seq library per animal per brain region (one biological replicate per library). Nuclear RNA was extracted with the RNeasy Mini Kit (74104, Qiagen) and RNase-Free DNase Set (79254, Qiagen). RNA-seq libraries were constructed using a modified Smart-seq3 protocol (47, 48) combined with a tagmentation-based approach adapted from ATAC-seq, employing custom annealed P5/P7 adapters loaded onto Tn5 transposase (Diagenode). Tagmented cDNA was PCR-amplified with KAPA HiFi ReadyMix and barcoded primers. Library fragment size was checked using the High Sensitivity DNA Analysis Kit (5067-4626, Agilent) on a TapeStation (G2992AA, Agilent), and primer-dimers were removed with SPRIselect beads. Libraries were quantified with the KAPA Library Quantification Kit (4824, Roche), pooled, and sequenced on an Illumina NovaSeq X Plus (paired-end 150 nt, ~15 million reads/library). Sex composition of RNA-seq samples per condition and region is provided in **Supplementary Table 1**.

#### 4.6.2 RNA-seq data processing and differential expression analysis

Sequencing reads were trimmed for adapters and quality using Trim Galore (v0.6.7) and aligned to the *Mus musculus* mm10 reference genome with STAR (v2.7.10a) (49). Gene-level counts were obtained using STAR with --quantMode GeneCounts and validated with HTSeq-count (v0.12.4) (50) against the GENCODE vM25 annotation. Genes with a total count ≤10 across all samples were removed prior to analysis. Differential expression was performed using DESeq2 (v1.40.2) (51) separately for each brain region; the design formula was ~sex + condition (four-level: SAL-SAL, MOR-SAL, SAL-LPS, MOR-LPS). Litter was not included as a covariate because it is nested within perinatal condition (see §4.1). The statistical unit is the individual animal. Pairwise contrasts were performed between MOR–SAL vs. SAL–SAL and MOR–LPS vs. SAL–LPS. Significance was defined at FDR < 0.05 (Benjamini–Hochberg). Significant DEGs were divided into upregulated (log_2_FC > 0) and downregulated (log_2_FC < 0) subsets. Functional enrichment of Gene Ontology biological process terms was performed using clusterProfiler (v4.8.3) (52).

### 4.7 Transcription factor regulatory network inference (Taiji)

Transcription factor (TF) regulatory influence was assessed using Taiji (v1.2.0) (53), Taiji constructs weighted TF–target gene networks by integrating two independent evidence sources: (i) presence of a TF binding motif within H3K27ac-marked enhancer regions proximal to target gene loci, and (ii) positive correlation between TF and target gene expression across biological replicates within each condition. TF influence was quantified as a personalized PageRank score reflecting network centrality; raw PageRank scores were z-score normalized per condition (mean=0, SD = 1) and capped at ±2 to limit outlier influence. Group-specific TFs (GS-TFs) were defined as TFs with condition-elevated PageRank z-scores identified by unpaired t-test (p < 0.05, |log_2_FC| ≥ 0.5) in one condition versus all others, with an additional RNA expression filter requiring detectable expression (>0 counts in ≥20% of samples within the target group). High-confidence TF–target edges were retained using a 98th percentile edge weight filter combined with an absolute Pearson expression correlation >0.3; the top 25 targets per TF were retained for network visualization. TF–target edge weights were compared between conditions using Wilcoxon rank-sum tests (p < 0.05, |Δweight| > 0.5); the rewiring index was calculated as the mean absolute change in edge weight across all significantly rewired targets. Taiji required matched QC-pass RNA-seq and ChIP-seq libraries; the hypothalamus analysis used 30 samples (SAL-SAL n=3, MOR-SAL n=8, SAL-LPS n=9, MOR-LPS n=10; sex composition provided in **Supplementary Table 1**).

### 4.8 Computational environment

All analyses were performed on a high-performance computing cluster (CentOS Linux, Slurm scheduler). Preprocessing and alignments were executed with Bash-based pipelines. Statistical analyses were conducted in R v4.3.2.

### 4.9 Cytokine measurements

Levels of select cytokines of interest were measured in the hypothalamus and spleen in the early postnatal period and in adulthood (at baseline and following LPS administration). The tissue samples were mechanically homogenized in 150-180 μL of phosphate-buffered saline (PBS) at pH 7.2, and an equal volume of Cell Lysis Buffer 2 (R&D Systems, Minneapolis, MN) was added. The samples were incubated for 30 minutes at room temperature with gentle shaking, then centrifuged at 13,000 g for 20 minutes at 4 °C. The supernatants were removed to a fresh 1.5 mL Eppendorf tube and immediately assayed by the Pierce BCA Protein Assay (Thermo Scientific, Rockford, IL) to obtain their mg total protein/ml values. The extracts were stored at -80 °C. A custom Milliplex^®^ MAP Mouse Cytokine/Chemokine Magnetic Bead Panel Luminex^®^ assay (MilliporeSigma, Burlington, MA) containing analytes for IL-15, IFNγ IL-1β, IL-2, IL-6, IL-10, IL12p70, IL-17A, CCL2/MCP-1, and TNFα was used. All extracts were run in duplicate, and the assay was run according to the kit enclosed instructions. The assay was read on a BioRad BioPlex^®^100 Luminex^®^ reader (BioRad Laboratories, Hercules, CA), using BioPlex^®^ Manager 6.1 software. The cytokine concentration values obtained, at pg/ml extract, were divided by their respective mg total protein/ml values to obtain pg/mg total protein values for each analyte.

### 4.10 Body temperature

Mice were exposed to the morphine exposure paradigm as previously described, then aged into adulthood without any further drug exposure. At 8–12 weeks, several physiologic tests were conducted. Telemetry implants (G2 E-Mitter, VitalView; STARR Life Sciences) were surgically implanted into the abdominal cavity of mice and were used for continuous monitoring of core body temperature. Mice were allowed to recover for a week before testing. Mice were single housed throughout testing. Data were collected for 24 hours following the start of the dark cycle. Collected data was binned into hour long segments for data analysis. Analysis was completed using VitalView Telemetry software (STARR Life Sciences).

### 4.11 Activity and sleep measurements

Mice were individually housed in fresh cages at the start of their light cycle. Their activity and sleep were monitored continuously using the COMPASS system, which employs overhead infrared sensors to detect movement. Activity was scored from 0–100 for every 10-second bin, representing the percentage of time spent moving. Activity score was calculated by averaging activity score for both light and dark cycles. Periods of extended inactivity (≥40 seconds) were classified as sleep, a metric validated against EEG recordings (54). A custom Python script identified these sleep bouts from the raw data. Sleep measurements were binned by light cycle and added together to determine cumulative sleep time for all light cycles.

### 4.12 Glucose tolerance test and Insulin tolerance test

In the GTT, mice were fasted for 16 hours (5:00 pm to 9:00 am) then given an i.p. injection of 2 mL/kg of 20% glucose. Blood glucose measurements were taken at 15 min, 30 min, 60 min, 90 min, and 120 min following injection. In the ITT, mice were given an i.p. injection of 1 U insulin/kg. Blood glucose measurements were taken at 15 min, 30 min, 60 min, 90 min, and 120 min following injection.

### 4.13 LPS administration and assessment of sickness behavior

A separate cohort of mice underwent the perinatal opioid exposure paradigm as previously described, then aged into adulthood without any further drug exposure. At 8–12 weeks, these mice were used to assess response to an immune challenge. To measure core body temperature, mice were surgically implanted with telemetry devices as previously described. After 2 days of recovery, mice were singly housed in a new room for analysis with COMPASS software and acclimated for 3 days prior to testing. Lipopolysaccharide (LPS) derived from *E. coli* O55:B5 (mdbioproducts; MDLPS5-0) was reconstituted using sterile 1x PBS. Mice were given an injection of LPS (2 mg/kg, i.p.) or saline approximately one hour into their dark cycle. A subset of mice was killed 2 hours after LPS administration to assess acute immune response. Remaining mice were used to study systemic immune response (temperature, sleep, activity) for 3 days following LPS injection. Body temperature was assessed using VitalView software, and sleep and activity levels were measured using the COMPASS system, as described above.

For overview of all experiments, see **Supplementary Figure 1**.

### 4.14 Statistical analysis

Results are expressed as mean ± SEM. The individual animal is the statistical unit for all analyses; technical processing steps do not constitute independent biological replicates. Litter was not included as a model covariate in sequencing analyses because it is nested within perinatal condition (see 4.1). The comparability among experimental conditions was assessed by a two-tailed unpaired or paired Student’s t-test, one-way analysis of variance (ANOVA), or two-way ANOVA as appropriate. For analysis of body temperature, GTT, and ITT, area under the curve (AUC) was calculated, followed by an unpaired t-test. When F achieved minimal statistical significance, the Tukey *post hoc* test was used for multiple comparisons. For all experiments, data were considered significant if p < 0.05. Outliers were determined using the Grubb’s Test (GraphPad Prism), with alpha < 0.05 used as cutoff for outliers. Dams were randomly assigned to receive morphine or saline. Experimenters were blinded to treatment groups during all data analysis. Males and females were combined for all sequencing studies but analyzed separately for all other molecular and behavioral studies. Data analysis was performed using Prism software (GraphPad Prism Version 10.2.2, Boston, MA, USA).

## Data Availability

ChIP-seq and RNA-seq data have been submitted to GEO under accessions GSE332794 (H3K27ac ChIP-seq) and GSE332798 (RNA-seq). All other data are available in the main text or the **Supplementary Materials**.
