# Supplementary material for: Perinatal morphine exposure induces chromatin and transcriptomic remodeling to alter immune and metabolic function
Source: Front Immunol. 2026 Jul 6;17:1835359. doi: 10.3389/fimmu.2026.1835359 (PMC13381623; doi:10.3389/fimmu.2026.1835359)
Supplement: Supplementary file 1 [file DataSheet1.pdf]

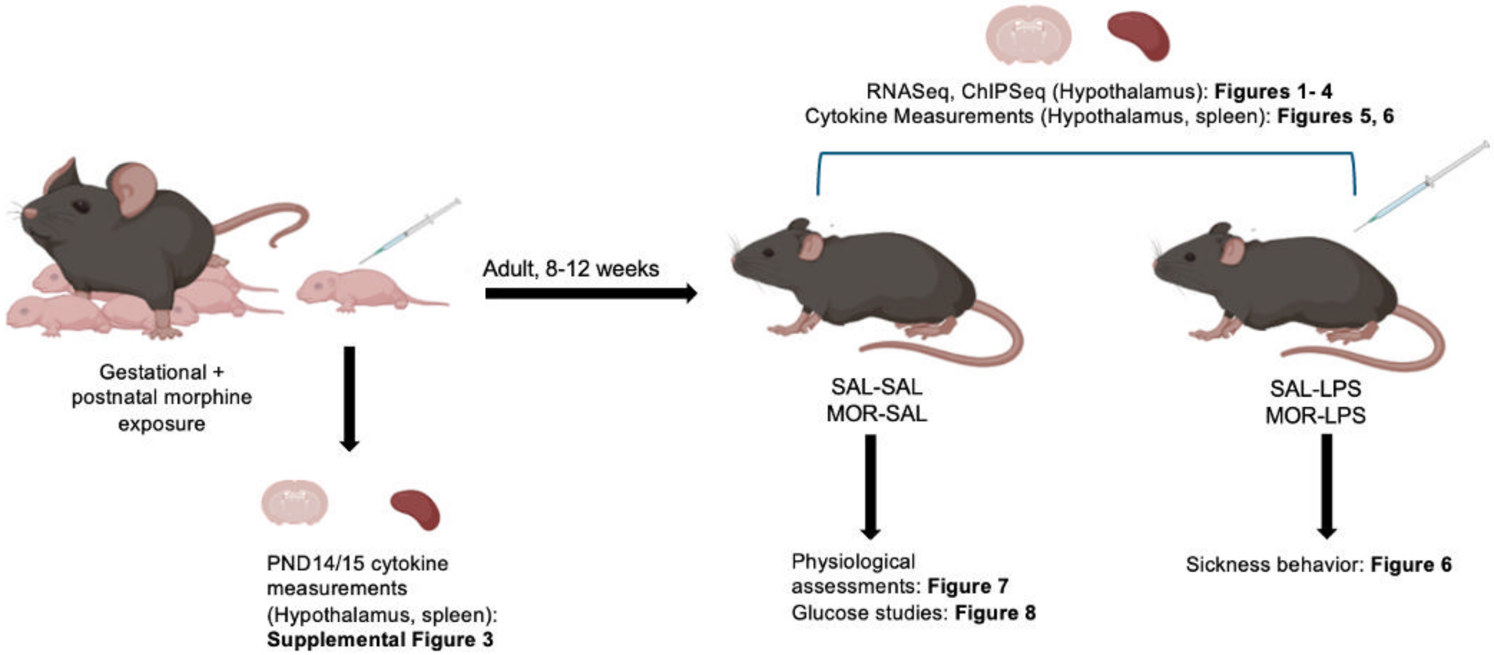

Supplemental Figure 1: Experimental timeline

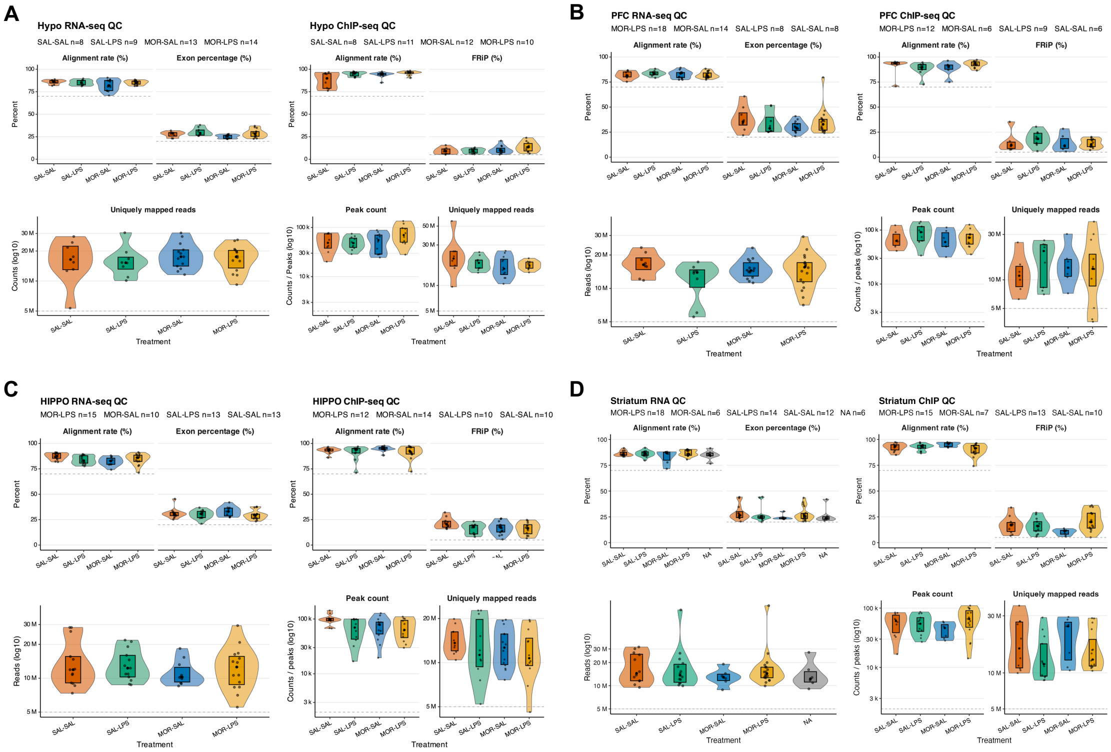

E

All regions: H3K27ac DiffPeaks (FDR < 0.05, |Fold| > 0.25, Promoter +/-1000bp)

region	contrast	FDR<0.05_Total	FDR<0.05_Enhancer	FDR<0.05_Promoter
Hippocampus	Baseline: MOR-SAL vs SAL-SAL	0	0	0
Hippocampus	LPS challenge: MOR-LPS vs SAL-LPS	0	0	0
Hypothalamus	Baseline: MOR-SAL vs SAL-SAL	67	49	18
Hypothalamus	LPS challenge: MOR-LPS vs SAL-LPS	2043	1361	682
PFC	Baseline: MOR-SAL vs SAL-SAL	0	0	0
PFC	LPS challenge: MOR-LPS vs SAL-LPS	0	0	0
Striatum	Baseline: MOR-SAL vs SAL-SAL	0	0	0
Striatum	LPS challenge: MOR-LPS vs SAL-LPS	648	383	265

F

All regions: DEGs (padj < 0.05, |log2FC| > 1)

region	contrast	FDR<0.05_Total	FDR<0.05_Up	FDR<0.05_Down
Hippocampus	Baseline: MOR-SAL vs SAL-SAL	5	4	1
Hippocampus	LPS challenge: MOR-LPS vs SAL-LPS	80	65	15
Hypothalamus	Baseline: MOR-SAL vs SAL-SAL	353	64	289
Hypothalamus	LPS challenge: MOR-LPS vs SAL-LPS	28	22	6
PFC	Baseline: MOR-SAL vs SAL-SAL	0	0	0
PFC	LPS challenge: MOR-LPS vs SAL-LPS	1	1	0
Striatum	Baseline: MOR-SAL vs SAL-SAL	0	0	0
Striatum	LPS challenge: MOR-LPS vs SAL-LPS	75	75	0

Supplementary Fig. 2: Quality control metrics and region-specific molecular responses across experimental conditions.
(A–D) Quality control metrics for bulk RNA-seq and H3K27ac ChIP-seq libraries across four brain regions: hypothalamus **(A)**, prefrontal cortex **(B)**, hippocampus **(C)**, and striatum **(D)**. For RNA-seq, violin plots show alignment rate (%), exon percentage (%), and uniquely mapped read counts per sample. For H3K27ac ChIP-seq, alignment rate (%), fraction of reads in peaks (FRiP, %), peak counts, and uniquely mapped read counts are shown. Each point represents an individual biological replicate, grouped by experimental condition (SAL–SAL, MOR–SAL, SAL–LPS, MOR–LPS). Distributions indicate comparable sequencing quality and library complexity across conditions within each region.
(E) Summary of differential H3K27ac peak counts across all regions. The table reports the total number of differential peaks (FDR < 0.05, |fold change| > 0.25), stratified by enhancer-associated and promoter-associated peaks (±1 kb from transcription start sites), for baseline (MOR–SAL vs SAL–SAL) and immune challenge (MOR–LPS vs SAL–LPS) contrasts.
(F) Summary of differentially expressed genes (DEGs) across all regions identified by RNA-seq (adjusted $p < 0.05$, $|\log_2FC| > 1$). The table reports total, upregulated, and downregulated DEGs for baseline and immune challenge contrasts.

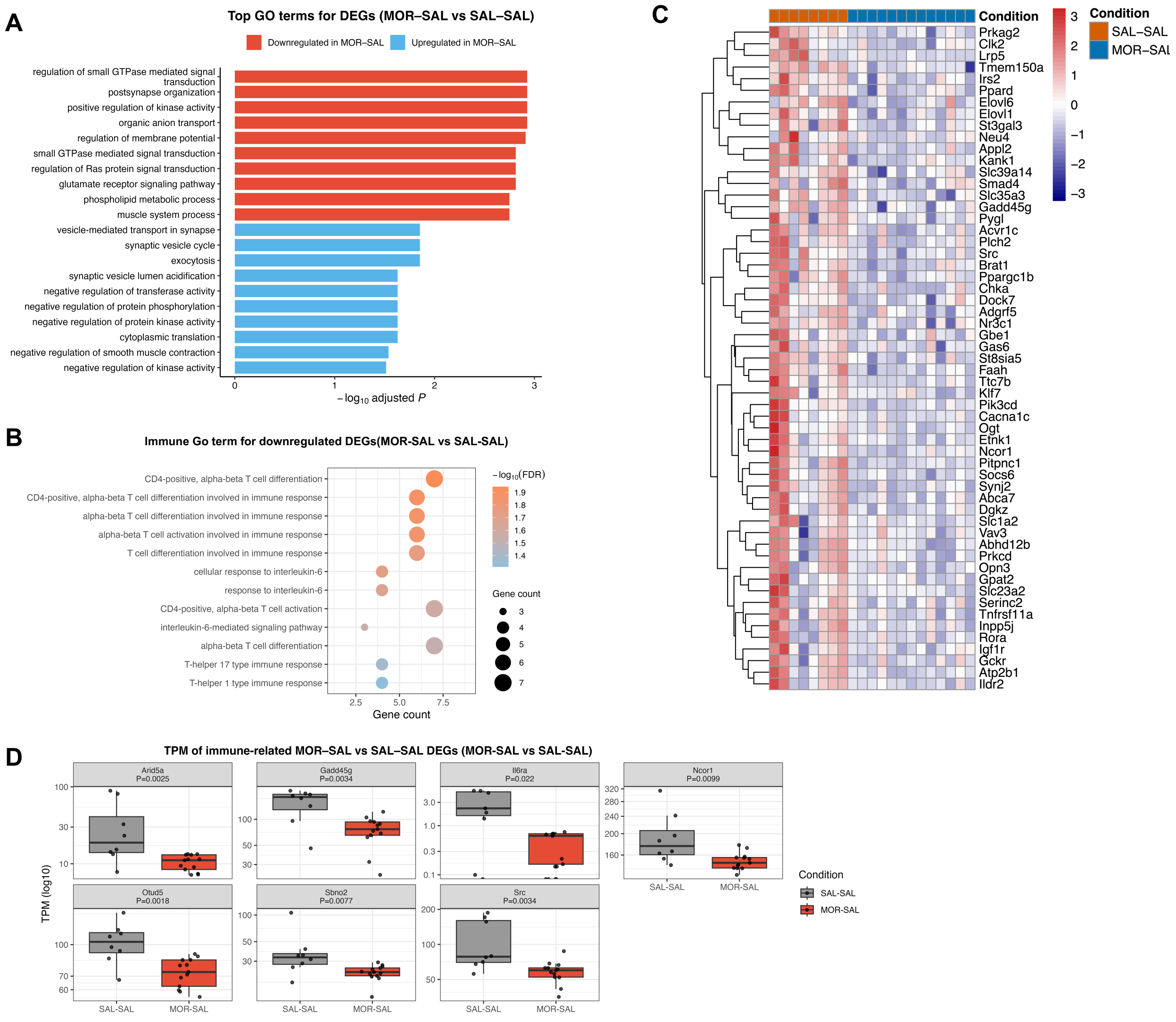

Supplementary Fig. 3: Early-life morphine suppresses baseline neuro-immune and metabolic transcriptional programs
(A) GO enrichment of DEGs in MOR–SAL vs SAL–SAL hypothalamus.
Upregulated genes (red) are enriched for neuronal and vesicle-transport pathways, whereas downregulated genes (blue) highlight loss of kinase regulation and cytoskeletal programs.
(B) Immune pathways downregulated in MOR–SAL animals.
GO terms show coordinated repression of T-cell differentiation and activation modules, IL-6–responsive programs, and Th1/Th17 signaling. Dot size reflects gene count; color indicates $-\log_{10}$(FDR).
(C) Heatmap of metabolism-related DEGs altered by early-life morphine exposure.
Expression of significantly dysregulated metabolic genes (rows) across biological replicates (columns) in SAL–SAL and MOR–SAL animals. Z-score–scaled TPM values highlight coordinated down-regulation of lipid, glucose, and thermogenic regulatory genes in MOR–SAL animals. Hierarchical clustering shows a coherent metabolic module disrupted by early-life morphine exposure.
(D) Representative immune regulators repressed in MOR–SAL.
Boxplots show reduced expression of Arid5a, Gadd45g, Il6ra, Otud5, Sbno2, Src, and Ncor1 in MOR–SAL relative to controls. Together, these analyses indicate that early-life morphine establishes a transcriptionally suppressed immune state prior to LPS challenge.

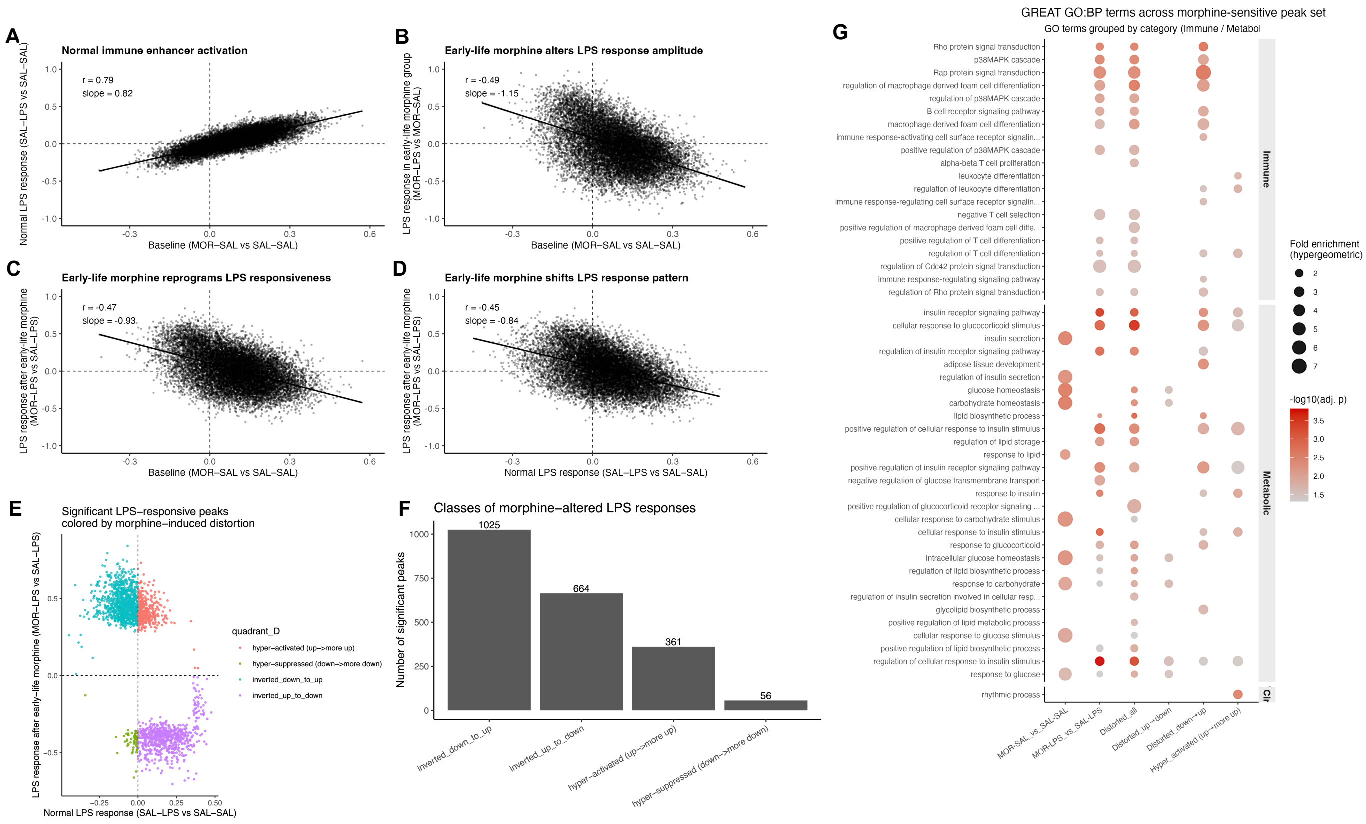

Supplementary Figure 4: Early-life morphine exposure distorts hypothalamic enhancer LPS responsiveness.
(A) Relationship between baseline enhancer acetylation imprint (MOR–SAL vs SAL–SAL; x-axis) and the normal LPS response (SAL–LPS vs SAL–SAL; y-axis), showing a strong positive association for immune-responsive enhancers.
(B) Comparison of baseline imprint (MOR–SAL vs SAL–SAL) with the LPS response measured within the morphine group (MOR–LPS vs MOR–SAL), revealing a negative correlation in which enhancers with higher baseline acetylation tend to show weaker or opposite LPS responses a[illegible]orphine, indicating a redistribution of LPS responsiveness rather than uniform gain.
(C) Scatter plot of baseline imprint (MOR–SAL vs SAL–SAL) versus the morphine-modified LPS contrast (MOR–LPS vs SAL–LPS), showing that morphine-imprinted enhancers are preferentially driven toward attenuated or inverted LPS responses.
(D) Direct comparison of normal versus morphine-altered LPS responses (SAL–LPS vs SAL–SAL on the x-axis; MOR–LPS vs SAL–LPS on the y-axis) demonstrating widespread pattern-level reprogramming, with a strong negative correlation indicative of response inversions across hundreds of enhancer regions.
(E) Significant LPS-responsive peaks (FDR < 0.05, |log2FC| > 0.25 in either condition) projected into the "normal vs morphine LPS response" space and colored by morphine-induced distortion type (hyper-activated, hyper-suppressed, inverted_up-to-down, inverted_down-to-up). This classification highlights the predominance of qualitative inversions rather than simple amplitude shifts.
(F) Barplot quantifying the distribution of morphine-altered enhancer response classes shown in (e), demonstrating that inversions represent the largest category of distortion.
(G) GREAT GO Biological Process enrichment for morphine-sensitive enhancer classes. Dot size reflects the number of associated peaks and color indicates fold enrichment. Immune-related pathways and stimulus-response gene programs show strong enrichment across distortion categories.

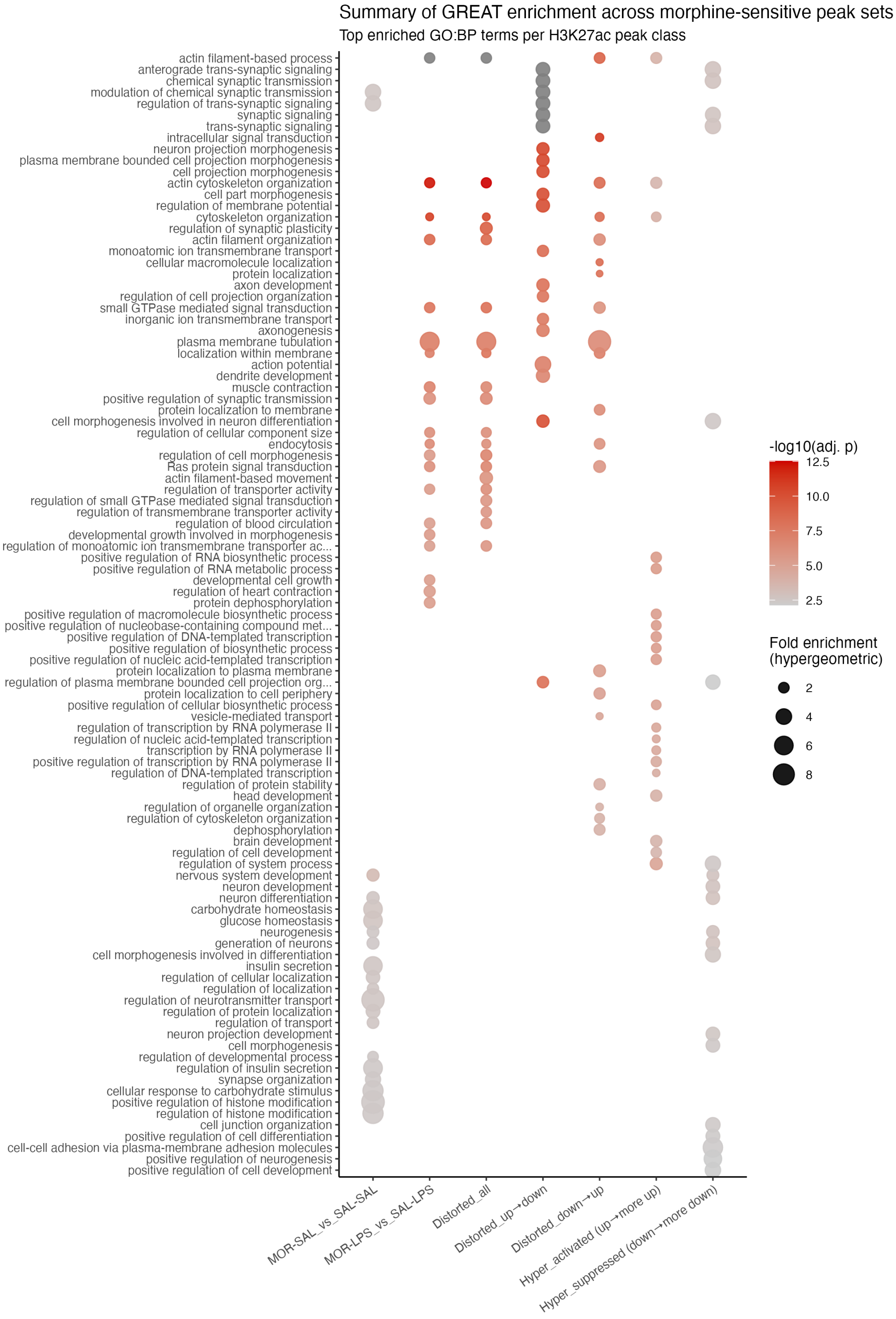

Supplementary Figure 5: Systems-level GREAT analysis of morphine-sensitive enhancer classes.
Bubble plot summarizing the top GREAT Biological Process terms enriched in H3K27ac peak classes altered by early-life morphine exposure. Columns represent (i) baseline MOR–SAL vs SAL-SAL, (ii) morphine-altered LPS responses (MOR–LPS vs SAL–LPS), and (iii) each distorted or amplitude-shift response subclass. Rows list the most significantly enriched GO:BP terms across all classes. Bubble color encodes –log10(adjusted p-value); bubble size reflects fold enrichment.

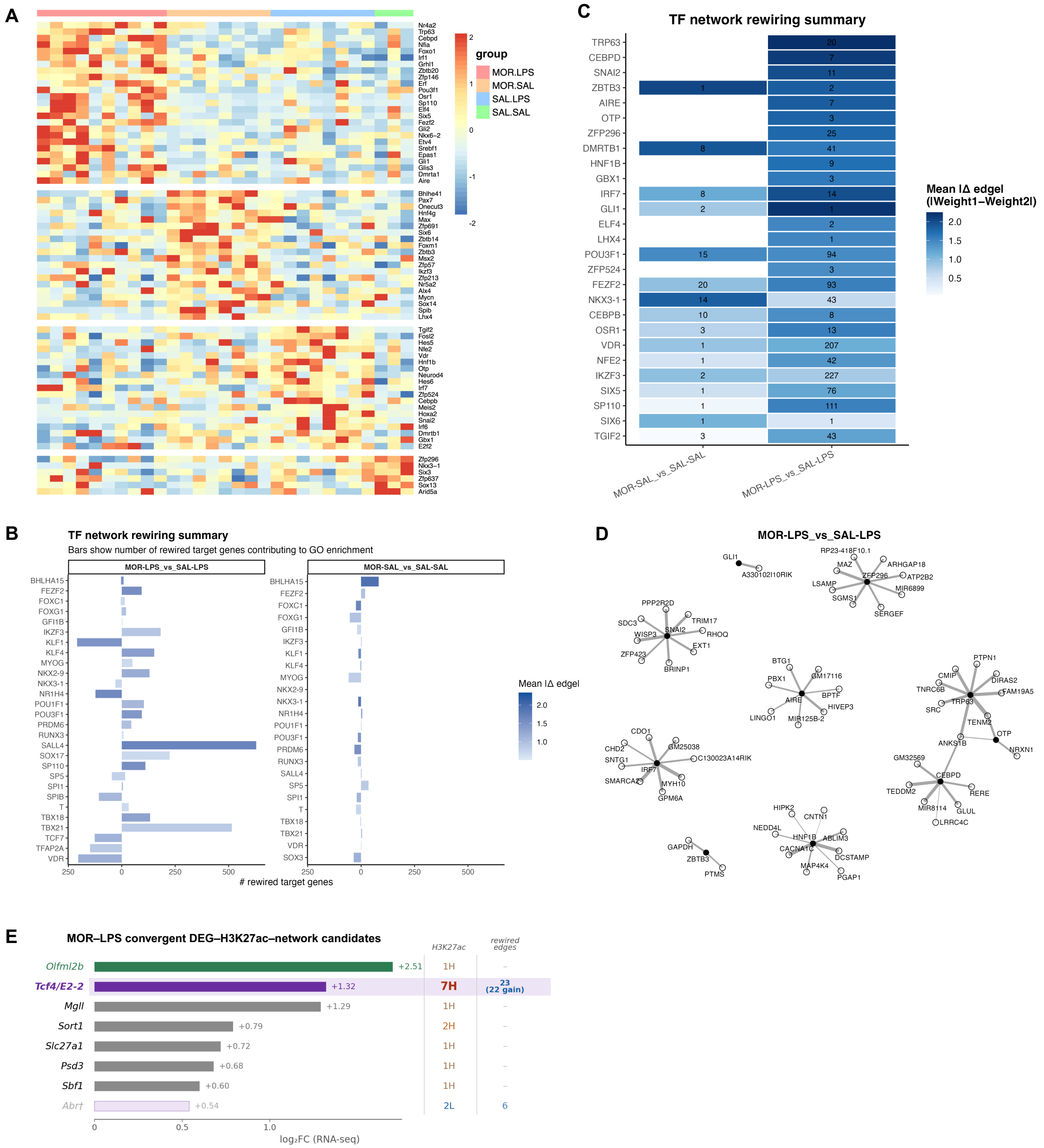

Supplementary Figure 6: Quantification and visualization of transcription factor network rewiring

(A) Heatmap of TF PageRank z-scores across all samples, shown for an expanded set of transcription factors. Samples are grouped by condition (SAL–SAL, MOR–SAL, SAL–LPS, MOR–LPS). Color scale indicates normalized PageRank centrality, highlighting condition-dependent shifts in TF network hierarchy.

(B) Summary of TF network rewiring for MOR–LPS vs SAL–LPS (left) and MOR–SAL vs SAL–SAL (right). Bars indicate the number of significantly rewired target genes per TF contributing to downstream Gene Ontology enrichment, with color denoting the mean absolute change in Taiji edge weight (|Δ weight|). Only TFs with significant rewiring are shown.

(C) TF-level rewiring summary comparing MOR–SAL vs SAL–SAL and MOR–LPS vs SAL–LPS. Heatmap shows the number of significantly rewired TF–target edges per TF, colored by the mean absolute change in edge weight across all rewired targets. Values are displayed within tiles.

(D) Representative TF–target subnetwork for the MOR–LPS vs SAL–LPS comparison, constructed from significantly rewired edges. Networks were filtered to retain high-confidence interactions (|Δ weight| > threshold and $P < 0.05$), illustrating condition-specific redistribution of regulatory connectivity. Nodes represent TFs and target genes; edges represent rewired regulatory interactions inferred from H3K27ac and RNA integration.

(E) Multi-layer summary of genes significant in both differential expression and H3K27ac analyses in MOR–LPS vs SAL–LPS. Bars show RNA-seq log2 fold change. The H3K27ac column indicates the number and direction of linked differential peaks (H, hyperacetylated; L, hypoacetylated). The rewired edges column indicates significantly altered upstream TF–target connections from Taiji analysis. Abr is marked as discordant because RNA is upregulated whereas associated H3K27ac peaks are hypoacetylated.

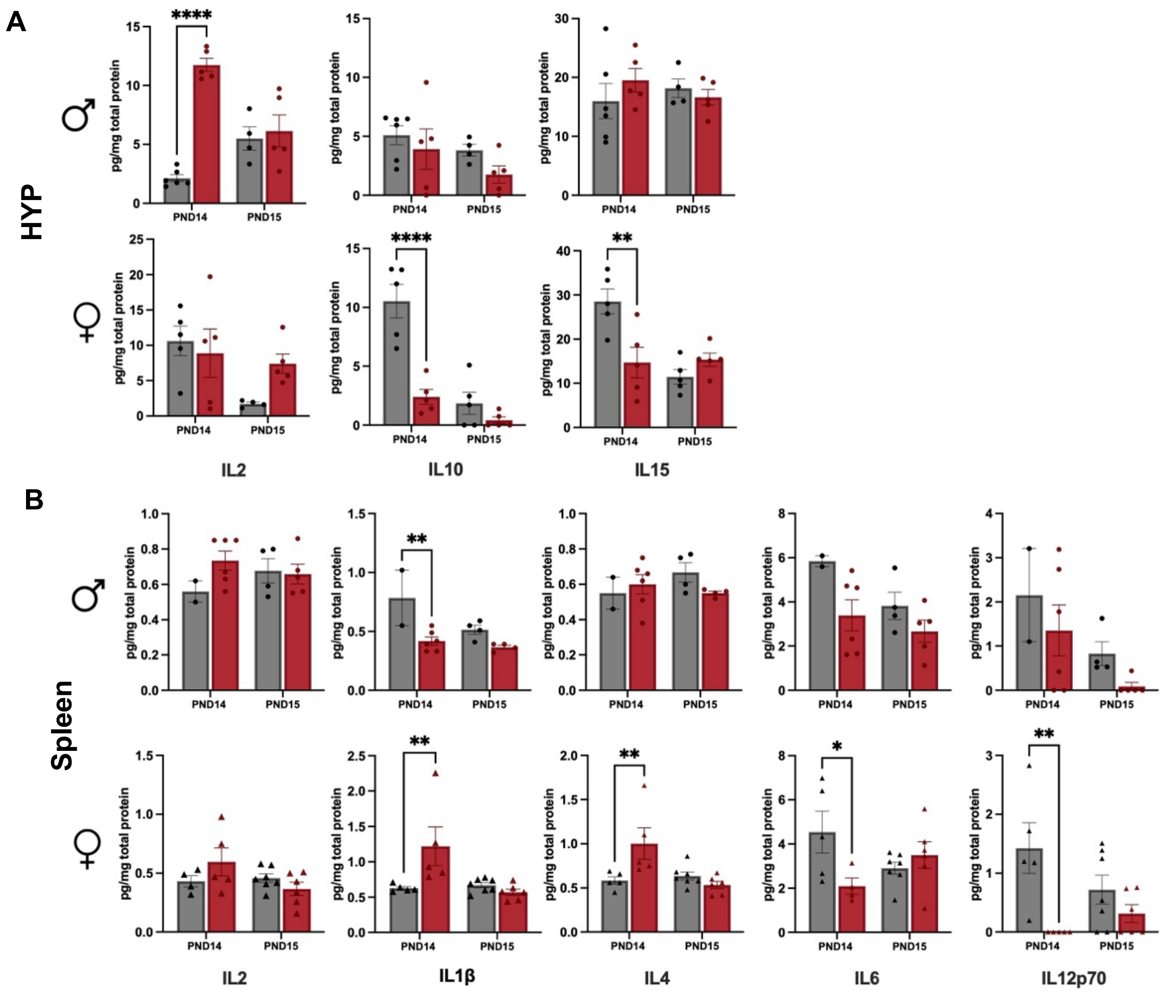

Supplemental figure 7: PND14/15 Cytokine measurements

(A) Graphs showing cytokine levels at PND14 and 15 in the hypothalamus in males (upper) and females (lower)

(B) Graphs showing cytokine levels at PND14 and 15 in the spleen in males (upper) and females (lower).

		IL15	IFNγ	IL1β	IL2	IL4	IL6	IL10	IL12p70	IL17a	CCL2	TNFα
Male	Spleen PND14	-	-	↓	-	-	-	-	-	-	-	-
	Spleen Adult No LPS	↓	-	↓	-	-	↓	↓	-	-	↓	-
	Spleen 2 hours post-LPS	-	↓	-	-	-	-	↓	-	Not detected	-	↓

		IL15	IFNγ	IL1β	IL2	IL4	IL6	IL10	IL12p70	IL17a	CCL2	TNFα
Male	Hypothalamus PND14	-	-	-	↑	-	-	-	-	-	-	-
	Hypothalamus Adult No LPS	↓	-	-	-	-	↓	↓	-	-	-	-
	Hypothalamus 2 hours post-LPS	-	Not detected	-	-	-	-	↓	-	↓	-	Not detected

Supplemental Table 1: Cytokine tables, male

		IL15	IFNγ	IL1β	IL2	IL4	IL6	IL10	IL12p70	IL17a	CCL2	TNFα
Female	Spleen PND14	-	-	-	↑	↑	↓	-	↓	-	-	-
	Spleen Adult No LPS	-	-	-	-	-	-	-	-	↓	-	-
	Spleen 2 hours post-LPS	-	-	↓	-	-	-	-	-	Not detected	-	-

		IL15	IFNγ	IL1β	IL2	IL4	IL6	IL10	IL12p70	IL17a	CCL2	TNFα
Female	Hypothalamus PND14	↓	-	-	-	-	-	↓	-	-	-	-
	Hypothalamus Adult No LPS	↓	-	-	-	-	-	↓	-	-	-	-
	Hypothalamus 2 hours post-LPS	-	Not detected	↑	-	-	-	-	-	-	-	Not detected

Supplemental Table 2: Cytokine tables, female
